# Discovery of antimicrobials by massively parallelized growth assays (M*e*^x^)

**DOI:** 10.1038/s41598-022-07755-7

**Published:** 2022-03-08

**Authors:** Philipp Koch, Steven Schmitt, Mathias Cardner, Niko Beerenwinkel, Sven Panke, Martin Held

**Affiliations:** 1 Bioprocess Laboratory, Department of Biosystems Science and Engineering, ETH Zurich, Basel, Switzerland; 2 Computational Biology, Department of Biosystems Science and Engineering, ETH Zurich, Basel, Switzerland; 3 SIB Swiss Institute of Bioinformatics, 4058 Basel, Switzerland

**Keywords:** Peptides, Biotechnology, Drug discovery, Drug screening, Data mining, High-throughput screening, Bioinformatics, High-throughput screening, Mechanism of action, Natural products, Peptides, Antimicrobials, Bacteria

## Abstract

The number of newly approved antimicrobial compounds has been steadily decreasing over the past 50 years emphasizing the need for novel antimicrobial substances. Here we present M*e*^x^, a method for the high-throughput discovery of novel antimicrobials, that relies on *E. coli* self-screening to determine the bioactivity of more than ten thousand naturally occurring peptides. Analysis of thousands of *E. coli* growth curves using next-generation sequencing enables the identification of more than 1000 previously unknown antimicrobial peptides. Additionally, by incorporating the kinetics of growth inhibition, a first indication of the mode of action is obtained, which has implications for the ultimate usefulness of the peptides in question. The most promising peptides of the screen are chemically synthesized and their activity is determined in standardized susceptibility assays. Ten out of 15 investigated peptides efficiently eradicate bacteria at a minimal inhibitory concentration in the lower µm or upper nm range. This work represents a step-change in the high-throughput discovery of functionally diverse antimicrobials.

## Introduction

Natural compounds are fundamental for drug discovery as they provide the biological relevance and structural diversity required to identify drug-like pharmacophores^1^. Owing to their high structural complexity and their ability to penetrate tissues and membranes, peptides are becoming increasingly important for many therapeutic areas^2^. Especially antimicrobial therapies have a very strong demand for novel compounds due to rising antimicrobial resistance^3^. Although about 3000 antimicrobial peptides have already been discovered, advances in genome sequencing and mining provide an ever-increasing number of peptides with elusive functions^4,5^.

Large peptide libraries can be screened for antimicrobial activity using bacteria self-screening protocols. Here, peptides are expressed from their encoding DNA template and then accumulate either in the cytosol, the periplasm or at the bacterial surface^6^. If antimicrobial, their expression negatively impacts the proliferation rate or survival of the expressing cell. Sequencing of the peptide-encoding DNA of such impaired cells allows for the identification of antimicrobials in large pools of uncharacterized peptides. However, previous self-screening approaches failed to deliver large fractions of highly active peptides, or were unsuited for the screening of big libraries^7–9^. Thus, novel high-throughput screening methods are urgently needed.

We gathered naturally-encoded peptides from peptide and genomic sequence databases and assayed them for antimicrobial activity using massively parallelized growth assays (M*e*^x^). Combined, the method delivered a rich collection of functionally diverse and highly active antimicrobial peptides.

## Results

**Figure 1:**
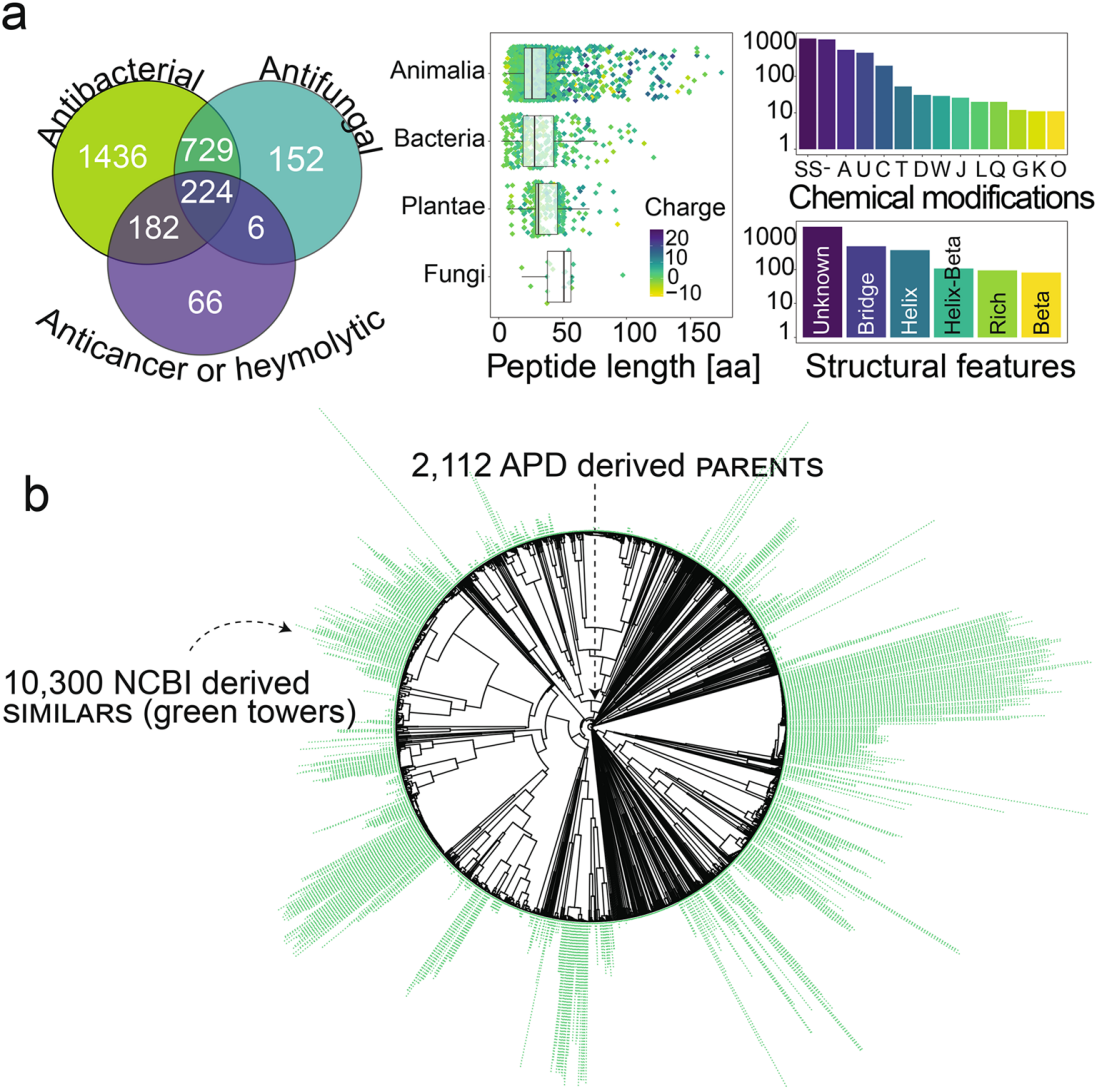
Generation of antimicrobial peptide library. (**a**) Biological diversity of parents. parents are derived from the APD. They have experimentally proven biological activity, e.g. antibacterial (Gram-negative and/or Gram-positive bacteria), antifungal, or anticancer or hemolytic, originate from species of various kingdoms of life, and differ considerably by length, charge, chemical modification (among others: *SS* disulfide bridges, *A* amidation, *U* terminal Rana box (disulfide bridge at C-terminus), *C* backbone cyclization, *T* thioether bridges, *D*-amino acids, *W* dehydration, *J* sidechain cyclization, *L* lipidation, *Q* terminal glutamate, *E* acetylation, *G* glycosylation, *K* hydroxylation, – no modification reported), and 3D-structure (*Beta* beta-sheet, *Bridge* disulfide bond, *Helix* alpha-helix, *Helix-Beta* alpha-helix and beta-sheet, *Rich* rich in unusual amino acids, *Unknown* no reported structure). (**b**) Sequence distances of the complete peptide library. Pairwise sequence distance between 2112 parents (BLOSUM62) as a basis for hierarchical clustering. similars found using *tblastn* for each parents’ search query are stacked as towers on the tips of the dendrogram.

We first designed a library of naturally-encoded peptides. For this, we collected the amino acid sequences of 3063 peptides with already experimentally validated activity (“Parents” from here on) from the antimicrobial peptide database (APD) (Fig. 1a)^4^. Notably, Parents differed considerably with respect to the host from which they were derived, length, physiochemical properties, chemical modifications, 3D-structure, and sequence (Fig. 1a/b). Next, we applied *tblastn* on the translated nucleotide databases accessible through the NCBI using the amino acid sequence of the Parents as queries^10^. This search yielded 36,898 amino acid sequences with a similarity of ≥ 21.1% to the Parents (“Similars” from here on). Unlike the Parents, only very few of the Similars have been synthesized or experimentally tested. However, owing to their natural origin and similarity to the Parents, a fraction of the Similars is likely to display antimicrobial properties^11,12^. For technical reasons (Methods), we applied a cut-off of 42 amino acids in peptide chain length and selected Similars with at least 62.2% sequence similarity. In this way, a library of 2122 Parents and 10,300 Similars (Fig. 1b) was obtained. Examination of the final library indicated net charges from − 10 to + 15 and hydrophobicity of − 3.5 to 2.9 (GRAVY scale; Fig. S1). Additionally, we were able to allocate the origin of 7497 of these peptides to the kingdom animalia, 74 to fungi, 678 to bacteria, and 2485 to plantae (Fig. S2).

**Figure 2:**
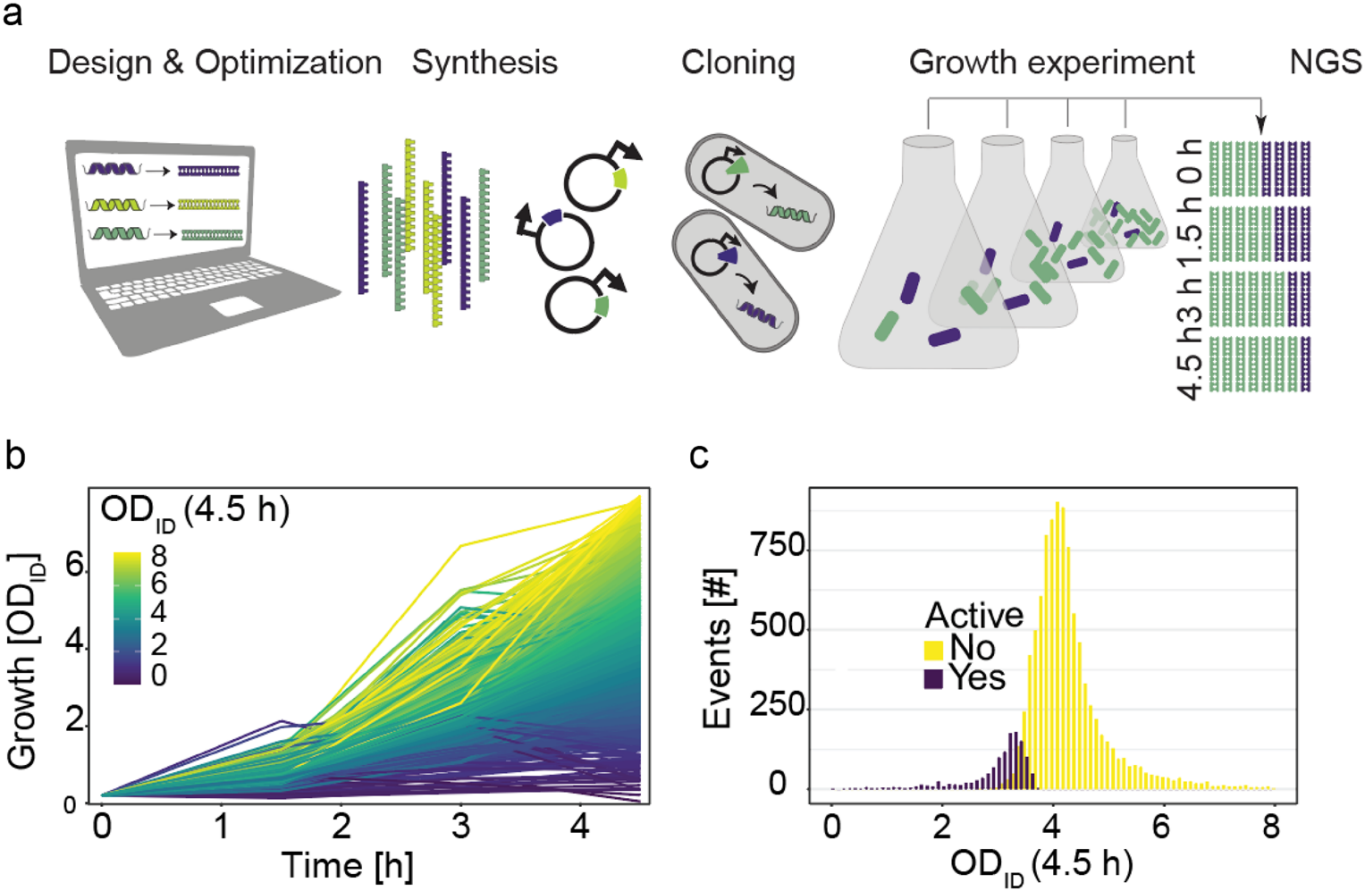
Screening of antimicrobial peptides using M*e*^x^. (**a**) M*e*^x^ workflow: Design and Optimization: Peptide sequences are reverse translated into *E. coli* codon-optimized nucleotide sequences. Synthesis: All peptide-encoding sequences are synthesized as oligonucleotides. Cloning: The sequences are inserted into plasmids. *E. coli* TOP10 is transformed with the generated peptide-encoding DNA library. Growth: Strains are incubated in shaking flasks, peptide expression is induced and plasmids are isolated. NGS: peptide-encoding DNA sequences are counted at four time points using NGS. (**b**) Growth curves of all 10,663 peptide-expressing strains, expressed as OD for a specific peptide-expressing strain (OD_ID_; average of n = 3). Coloring from yellow to dark blue indicates higher growth inhibitory effects based on OD_ID_ of last sampling point. Curves reaching a higher OD_ID_ than eight (0.7%) are omitted for clarity. (**c**) Growth inhibition at 4.5 h recorded for all 10,663 peptide-expressing strains. OD_ID_-values are recorded for each of the peptide-expressing strains and are averaged from three replicates. M*e*^x^-active peptides (purple) significantly (Wald's test, adj. *p* < 0.05) reduce the OD_ID_ of their expressing strain after 4.5 h while M*e*^x^-inactive peptides (yellow) fail to do so. Note that some candidates may also fail to reach statistical significance in the performed M*e*^x^-activity test due to low NGS read counts or high variance between replicates (Supplementary Table 2).

For M*e*^x^*,* we converted the peptides into corresponding oligonucleotides (Fig. S3), retrieved the latter as a pool after chemical synthesis on a microarray, and ligated the sequences into a plasmid on which their expression was controlled by the tightly regulated P_BAD_ promotor (Fig. 2a). We then transformed the model organism, *E. coli* TOP10, with the peptide-encoding DNA library. Using next-generation sequencing (NGS), we only identified 10,663 different peptide-encoding DNA sequences (listed by ID in File S1) in *E. coli* indicating sequence bias in the initial oligonucleotide pool (Fig. S4).

To assess the antimicrobial activity of the DNA-encoded peptides, we performed M*e*^x^ and generated growth curves for each of the 10,663 peptide-expressing *E. coli* strains. To do so, we inoculated three liquid cultures each with 500 million transformed cells, and induced peptide synthesis after four cell doublings (Fig. S5). Because the synthesis of an antimicrobial peptide should inhibit the growth of the expressing host, the propagation rate of the peptide-encoding DNA will also be reduced. Hence, we harvested bacteria at the time of induction as well as 1.5 h, 3.0 h, and 4.5 h post-induction and used NGS to count reads for each peptide-encoding DNA. To derive growth curves (Fig. 2b), we calculated the abundance of each strain (ID) using the respective NGS read counts and multiplied these with the measured cell concentration of the entire liquid cultures (OD) thereby obtaining an approximation of the strain-specific concentrations (OD_ID_) at each sampling point. Comparing OD_ID_ of all peptide-expressing strains after 4.5 h, we found that intracellular expression of 1240 peptides (11.6%) significantly inhibited the growth of their host (“M*e*^x^-actives” from here on; Wald’s test, p-value (*p*) < 0.05, adjusted for multiple testing (adj.); Fig. 2c). The remaining peptides did not show growth inhibition in M*e*^x^, likely because they are not antimicrobial at all or require chemical modifications not introduced in *E. coli*, could not access their (e.g. extracellular) target, or did not reach inhibitory concentrations due to limited mRNA or peptide stability.

Next, we confirmed that the intracellularly synthesized peptides also inhibited growth if the strains were grown individually. For this, we selected 110 peptide-expressing strains experiencing different levels of growth inhibition in M*e*^x^ and measured their growth in microtiter plate wells (Fig. S6a/b). As the growth curves recorded in M*e*^x^ and microtiter plates were comparable (Fig. S6c), we concluded that the complex dynamic of the M*e*^x^-culture did not bias the results.

Screening 10,663 peptides at once allowed us to address several research questions. Firstly, we sought to confirm that our approach of exploiting sequence similarities to known antimicrobial peptides indeed allowed us to identify antimicrobials. In fact, 1035 out of 1240 M*e*^x^-actives (83%) were Similars, i.e. peptides whose functions were not reported on the APD. A closer look revealed that for 310 inactive Parents we found at least one active Similar. As an example, Parent Apo5 APOC1_667 APD_ (nomenclature: name of Parent on APD _ID Origin_), itself inactive, yielded 27 Similars of which one showed eight amino acid differences to the Parent and displayed antimicrobial activity (Fig. S7). We argue that the amino acids by which the inactive parent and the active similar differed were of high importance for activity and necessary for evading the abovementioned reasons for failed growth inhibition in M*e*^x^. Furthermore, 47 Parents spawned an overrepresentation of active Similars (Fisher’s exact test, adj. *p* < 0.05; Fig. S8). Examples include Myticin-B (21/31), which yielded 31 Similars, of which 21 were active, and PepG1 (11/11). This indicates that the respective peptide sequences have considerable plasticity and can accommodate multiple amino acid exchanges without losing activity. We argue that these peptides might be well suited for additional modifications performed for instance in the course of lead optimization programs^13^.

Secondly, we evaluated the phylogeny of the hosts from which the inhibitory peptides were derived. For this, all peptides of the library were grouped taxonomically based on their natural host. We then calculated the fraction (%) of M*e*^x^-actives within the ranks Kingdom and Class (Fisher’s exact test; Fig. S9). M*e*^x^-actives were significantly underrepresented (*p* < 0.05) among bacteria (8.5%), amphibians (7.7%), and mammals (10.3%) but overrepresented (*p* < 0.05) in insects (13.4%), birds (25%), ray-finned fishes (15.6%) and bivalves (31.8%). Since insects contain by far the most species in the animal kingdom, this indicates a huge and so far undiscovered pool of antimicrobials in insects.

Thirdly, as cationic and hydrophobic peptides frequently display antimicrobial activity, we wondered whether growth inhibition in M*e*^x^ was biased by the physiochemical properties of peptides^14^. However, linear regression analysis indicated no correlation of growth inhibition with hydrophobicity (correlation = 0.04) and charge (correlation = − 0.01; Fig. S10a). Furthermore, among the 47 Parents with overrepresented active Similars, there was no clear relationship between charge or hydrophobicity and growth inhibition (Fig. S10b). We thus conclude that growth inhibition in M*e*^x^-actives is driven by the specific antimicrobial activity of a peptide either damaging the cytoplasmic membrane or binding and inhibiting other cellular components.

**Figure 3:**
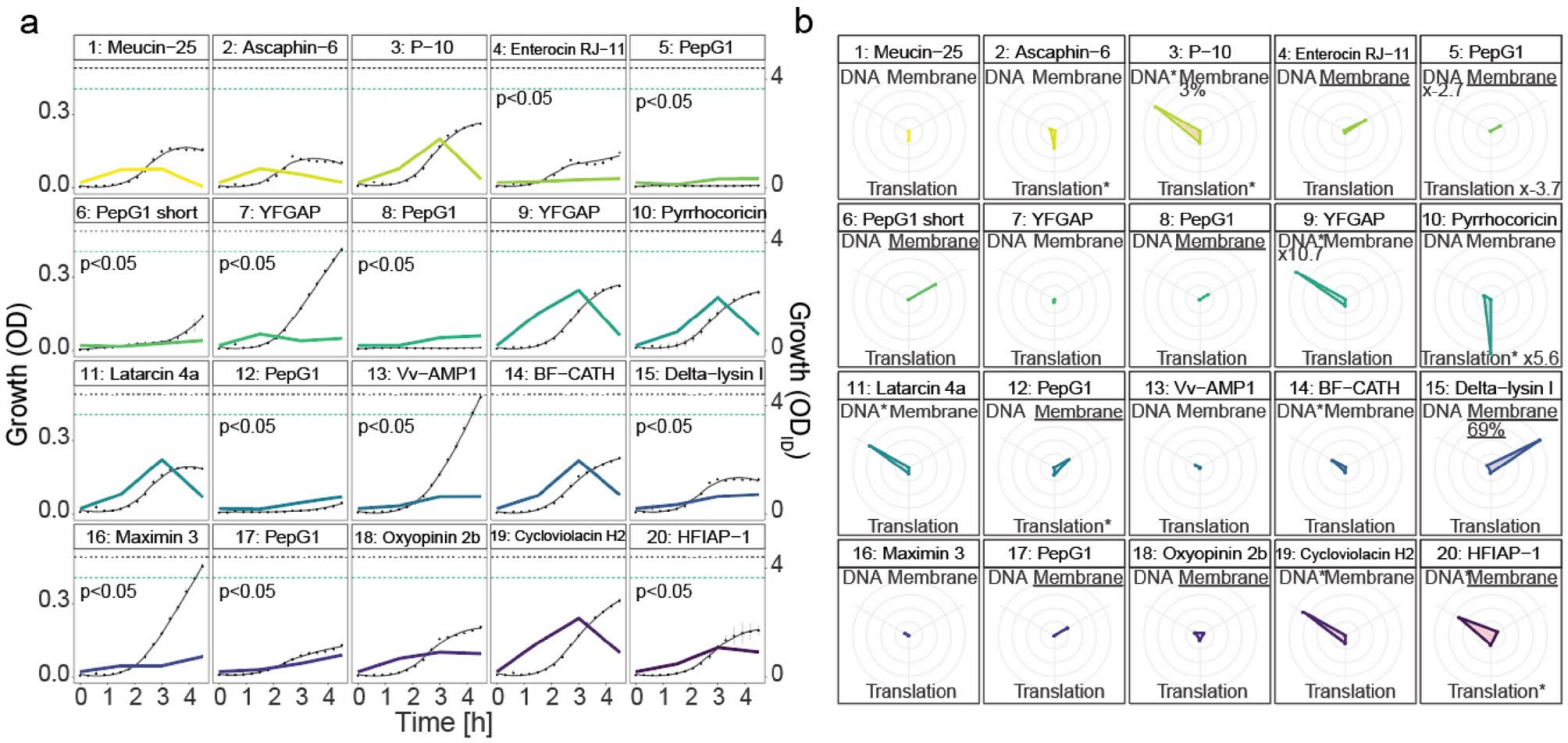
Characterization of the 20 most active peptides in M*e*^x^. (**a**) Growth curves of *E. coli* TOP10 cells each expressing on of the 20 most active (by OD_ID_ at 4.5 h) peptides. Colored lines are M*e*^x^-recorded growth curves (average of n = 3) determined via OD_ID_ approximation (header: ‘rank: parent name’). Black lines are growth curves (n = 3, error bars: 2σ) determined via OD measurement in microtiter plates of individually grown strains. Horizontal dashed lines, in black (OD) or colored in green (OD_ID_), show final values measured 4.5 h post-induction of a strain synthesizing the inactive control peptide HNP-1_3425 APD_ (obtained from Fig. S13). In each facet, we state if we obtain a *p* < 0.05 (Wald’s test) for significant growth inhibition after 1.5 h in M*e*^x^. (**b**) Potential mechanisms of action. Each radar plot shows the mean SOS-response (DNA; activation of the *recA* promoter; n = 3), translation inhibition (Translation; activation of the *cspA* promoter; n = 3), and membrane-damage (Membrane; PI stained cells in percent; n = 2) obtained after peptide expression in *E. coli* TOP10. Only the maximum and minimum values are reported in digits. The center represents values measured for the negative control peptide HNP-1_3425 APD_. Lower values are scaled to the center. Membrane damage is attributed if more than 10% of cells were PI-positive (underlined). For SOS and Translation, signals are reported relative to the signal obtained for the inactive control peptide HNP-1_3425 APD_. A significant increase (one-sided *t* test, adj. *p* < 0.05) compared to the inactive control is indicated by an asterisk (*).

To investigate peptides further, we characterized the 50 most growth inhibitory peptides as indicated by their rank in the M*e*^x^ screening (rank 1–50; 38 similars, 12 parents) (Fig. S11a–c). Initial tests were performed with two biosensor constructs, containing the *cspA* and *recA* promoters, which upon activation are indicative of translation impairment and DNA damage, respectively^15^. The results indicated translational impairment for 11 and DNA damage for 12 peptide-expressing strains (one-sided *t* test, adj. *p* < 0.05; Fig. 3b; Fig. S12), which suggests that these peptides target intracellular macromolecules. In fact, many peptides traverse the membrane(s) of bacteria without permeabilization and kill cells by binding or blocking intracellular macromolecules^16,17^. For example, Metalnikowin IIA_8984 APD_, Metalnikowin III_9011 APD_, known ribosomal inhibitors, and Pyrrhocoricin_7122 NCBI_, whose parent is also a ribosomal inhibitor, caused the strongest indication for translational impairment in our assay^18^. Next, we measured membrane damage by quantifying propidium iodide (PI) uptake. Expression of 11 peptides resulted in membrane damage, with the strongest damages observed for Delta Lysin I similars whose Parent is a membrane pore inducing bacteriocin from *Staphylococcus* (Fig. S12, Table S1). Interestingly, 11 out of 12 peptides that caused membrane damage significantly inhibited growth already after 1.5 h in M*e*^x^ (Wald’s test, *p* < 0.05) (Fig. 3a; Fig. S13) while for 25 peptides, and especially for those with putative intracellular targets, growth inhibition started only after 4.5 h (Fig. 3a; Fig. S13). Noteworthy, delay of the growth inhibition onset has been reported to be indicative of peptides that interact with an intracellular target^19^. We concluded that this effect could be observed in M*e*^x^ too, and hence reanalyzed all growth curves recorded for the M*e*^x^-actives. Growth was significantly inhibited after 1.5 h by 806 peptides (65%) pointing towards membrane damage (File S1) but only after 4.5 h in the case of the remaining 434 peptides, suggesting a high likelihood for reduced membrane damage, or the interaction with an intracellular target.

Next, we chemically synthesized 15 out of the 20 peptides that were found to be most growth inhibitory in M*e*^x^ and determined their minimal inhibitory concentrations (MIC) in both 100% Mueller–Hinton Broth (MHB) and 25% MHB, their membrane damaging capabilities in 100% MHB, and hemolytic activity when added to cells as a synthesized chemical (Table 1; Fig. S11).

**Table 1:** Summary of antimicrobial activity assays of the 20 most active peptides in M*e*^x^. Peptides for which a MIC could be determined are highlighted in bold. PepG1 similars (rank 6, 8, 12, and 17), and Cycloviolacin H2 (rank 19) were removed from this table, as the purification failed. Intracellular characterization is derived from the experiments summarized in Fig. 3b. Mean MIC-values are recorded (n = 3) in microtiter plate assays using chemically synthesized peptides against the screening strain *E. coli* TOP10. Membrane damage is reported at the peptide concentration, relative to the MIC, at which at least 25% of cells acquired externally added PI. (n = 2; Fig. S14a). Hemolysis as a percentage is related to full lysis after treatment with 2.5% Triton-X100 (n = 4; Fig. S15). MIC of ampicillin (control) against *E. coli* TOP10: 34 µm (100% MHB); 17 µm (25% MHB).

Rank	parent name	Peptide sequence	Origin	ID	Charge	GRAVY	Intracellular characterization	MIC [µm] 100%MHB	MIC [µm] 25%MHB	Membrane damage	Hemolysis at MIC | 16MIC
1	Meucin-25	VKLIQIRIWIQYVTVLQMFSMKTKQ	APD	11,598	+ 4	0.4	–	> 60	> 60		
**2**	**Ascaphin-6**	**GFKDWIKGAAKKLIKTVASSIANE**	**APD**	**9286**	**+ 3**	**− 0.1**	**Translation**	**0.8**	**1**	$\frac{1}{2}$ **MIC**	**0% | 1.4%**
**3**	**P-10**	**VSKIKKYLKYKDRI**	**APD**	**8942**	**+ 5**	**− 1**	**DNA; Translation**	**> 60**	**13**	**No damage**	**0.5% | 2.2%**
**4**	**Enterocin RJ-11**	**AIAKLVAKFGWPIVKKYYKQIMQFIGEGWAINKIIEWIKK**	**NCBI**	**3780**	**+ 6**	**0.2**	**Membrane**	**0.5**	**1**	$\frac{1}{4}$ **MIC**	**22.7% | 103.3%**
5	PepG1	MITISTMLQFGLFLIALIGLVIKLIELSIKK	NCBI	11,834	+ 2	1.6	Membrane	> 60	> 60		
**7**	**YFGAP**	**VKVGINGFGRIGRLVTRAAFQSKKVEIVAIND**	**NCBI**	**8112**	**+ 4**	**0.3**	**–**	**> 60**	**8**	**2MIC**	**0% | 3.3%**
**9**	**YFGAP**	**VKVGVNGFGRIGRLVTRAAFNSGKVEIVAIND**	**NCBI**	**8135**	**+ 3**	**0.4**	**DNA**	**> 60**	**5**	**2MIC**	**0% | 0.4%**
**10**	**Pyrrhocoricin**	**VDKGGYLPRPTPPRPVYR**	**NCBI**	**7122**	**+ 3**	**− 1.2**	**Translation**	**20**	**8**	**No damage**	**1.1% | 2.3%**
**11**	**Latarcin 4a**	**LKDKVKSMGEKLKQYIQTWKAKF**	**APD**	**5147**	**+ 5**	**− 1**	**DNA**	**8**	**5**	**2MIC**	**0.2% | 2.8%**
13	Vv-AMP1	RACESQSHRFKGTCVRQSNCAAVCQTE	NCBI	8053	+ 2	**− **0.7	–	> 60	> 60		
**14**	**BF-CATH**	**KRFKKFFKKLKKSVKKRAKKFFKKPRVIGVSIPF**	**APD**	**9639**	**+ 16**	**− 0.7**	**DNA**	**1**	**1**	**4MIC**	**2.1% | 4.8%**
15	Delta lysin I	MAADIISTIGDLVKWIIDTVNKFK	NCBI	3458	0	0.6	Membrane	> 60	> 60		
16	Maximin 3	TALKGAAKELASTYQH	NCBI	5468	+ 1	**− **0.4	–	> 60	> 60		
**18**	**Oxyopinin 2b**	**GKFSGFAKILKSIAKFFKGVGKVRKGFKEASDLDKNQ**	**APD**	**9690**	**+ 7**	**− 0.5**	**Membrane**	**0.5**	**0.5**	$\frac{1}{2}$ **MIC**	**0.7% | 63.4%**
**20**	**HFIAP-1**	**GWFKKAWRKVKHAGRRVLDTAKGVGRHYLNNWLNRYR**	**NCBI**	**4545**	**+ 10**	**− 1.1**	**Membrane, DNA; translation**	**0.5**	**0.5**	$\frac{1}{8}$ **MIC**	**4.1% | 18.9%**

Firstly, no MIC was obtained for five peptides; however, as four of these five were either Parents or derived from Parents known to be inactive against *E. coli* (Table S1), we believe that these peptides exerted activity in the cytosol if synthesized intracellularly but could not reach their target (e.g. the cytoplasmic membrane) when added as a chemical substance to the growth medium. Remarkably, 10 of the 15 peptides for which MICs were recorded, very efficiently inhibited the growth of *E. coli* (MICs 0.4–20 µm; mean = 3.7 µm; median = 1 µm), in a concentration range that could qualify as a starting point for drug development^20^. In four cases (P-10_8942 APD_, YFGAP_8112 NCBI_, YFGAP_8135 NCBI_ and Pyrrhocoricin_7122 NCBI_) the activity increased in diluted MHB, while for the remaining six peptides, a similar MIC was recorded in both 100%, and 25% MHB. We selected the most active Similar, HFIAP-1_4545 NCBI,_ and measured the activity against other clinically relevant Gram-negative and positive bacteria. Similar HFIAP-1_4545 NCBI_ inhibited growth of these strains (MICs: 0.4–5.6 µm; Table S2), which suggests a broad activity spectrum even though M*e*^x^ screening was performed in another host. These results indicated that although we screened the peptide library synthesized cytosolically, M*e*^x^-active peptides also strongly inhibited growth when added to cells externally and that the employed *E. coli* lab strain was suited well for the identification of peptides also active against other bacterial species.

Secondly, to evaluate the degree rely on damaging of membranes damage for the exertion of antimicrobial, we measured the uptake of PI, when adding the peptides four log_2_ concentration steps above and below their MIC. As complete lysis of both outer and inner membrane resulted in false negatives in PI measurement (see the decrease in PI signal when increasing the concentration of Melittin in Fig. S14), we also quantified the point at which both membranes were lysed by measuring the release of intracellularly expressed GFP. Only Ascaphin-6_9286 APD_, Enterocin RJ-11_3780 APD,_ Oxyopinine 2b_9690 APD_, and HFIAP-1_4545 NCBI_, showed strong membrane damage (> 25% PI-positive cells below MIC concentration) in a range of the membrane damaging peptide Melittin (Table 1; Fig. S14). For the remaining peptides, and especially for those with reported intracellular targets (Fig. 2b) and a delayed growth inhibitory effect in M*e*^x^ (Fig. 2a), no membrane damage could be detected at all, or occurred considerably above the MIC (Fig. S14). Hence, other mechanisms, such as blocking of protein translation as reported for the parent of Pyrrhocoricin_7122 NCBI_, must play a role in bacterial killing^18^. These results confirmed that the previously recorded intracellular characterization of the peptides can be a good indication for the activity of chemically produces peptides discovered in the M*e*^x^ assay.

Lastly, as hemolysis is a reliable and sensitive indicator for cytotoxicity assessment, we measured the hemolytic activity of all MIC active peptides^21,22^. Toxicity towards human erythrocytes (> 5% hemolysis compared to the Triton-X100 control) at the MIC was only observed for the membrane damaging peptide Enterocin RJ-11_3780 NCBI_, a known hemolytic staphylococcal toxin, and for the control Melittin (Table 1)^23^. Additionally, the membrane damaging peptides Oxyopinine 2b_9690 APD_, a known hemolytic spider toxin, and HFIAP-1_4545 NCBI_ displayed hemolytic activity at higher concentrations (Fig. S15; Table 1)^24^. All other peptides did not damage erythrocytes at the tested concentration. This suggests that most of the active peptides found in the M*e*^x^ screen were not cytotoxic thereby further corroborating the potential of the isolated hits for drug development.

## Discussion

We applied M*e*^x^ for the highly parallelized discovery and characterization of more than 10,000 structurally diverse, and naturally-encoded peptides (Fig. 1a/b; Fig. S1; File S1). Chemical production and microtiter plate based screening of a library of such high diversity and size, and containing peptides longer than 40 amino acids would have been very cost- and time-consuming. However, M*e*^x^ takes a shortcut by using in silico optimized and pooled oligonucleotides as templates for ribosomal peptide synthesis (Fig. S3) and intracellular activity assessment via monitoring the growth inhibitory effects (Fig. 2a/b).

Growth curves recorded by M*e*^x^ via NGS were comparable to those obtained for a few tested strains if grown compartmentalized in microtiter plates (Fig. S6). This indicated that pooling of the peptide-expressing strains did not bias the experimental outcome. Moreover, the high hit rate (10 out of 20 peptides) obtained for M*e*^x^-active peptides if synthesized as chemicals and tested in MIC assays under stringent CSLI- assay conditions (Table 1) corroborates the robustness of M*e*^x^. In addition, screening of naturally-encoded sequences delivered a large fraction of highly active peptides, by far exceeding the performance of other approaches selecting randomly or semi-randomly designed peptide libraries^7–9^. As naturally occurring peptides are preselected for biological activity, including antimicrobial activity, our results confirmed the advantage of screening sets derived from genomic databases^1,12,25^.

When extrapolating from our hit-rates (50% of M*e*^x^-actives were active in MIC assays using chemically synthesized peptides) to the entire library (1035 active similars and 205 active parents), we found more than 500 previously unreported, active antimicrobial peptides, derived from organisms of various biological classes (Fig. 2c; Fig. S9). Moreover, by analyzing growth curves of the peptide-expressing strains (displayed as OD_ID_, Fig. 3a; Fig. S13), including data indicative for intracellular stress response mechanisms (Fig. 3b; Fig. S12), and the status of the bacterial membrane integrity (Table 1, Fig. S14), we estimated in *E. coli* TOP10 about one-third of the active peptides most likely exert antimicrobial activity via the interaction with intracellular targets (see all in File S1). We want to point out that all data was generated using the weakened laboratory strain *E. coli* TOP10, and thus cannot be simply adapted to others strains of *E. coli* (e.g. clinical isolates), other Gram-negative, or even Gram-positive bacteria.

These results hence cast a fresh look on the field of antimicrobial peptides as only very few examples (< 50) of intracellularly active peptides are known^26^. We hypothesize that nature designed the peptides such that passage of bacterial membranes and binding to macromolecules is a frequently built-in feature. As the transition from the discovery pipeline to the patient is often hampered by the poor specificity of membrane damaging peptides in vivo, M*e*^x^ can be a valuable tool for the high-throughput discovery of peptides that do not rely on membrane damage for bacterial killing^27,28^.

Our indicated mechanism (peptides of rank 1–20 summarized in Table 1) correlate well with those reported for the parent peptides by others (Table S1). Membrane damage was reported for parents (of) Ascaphine-6, Enterocin RJ-11, Oxyopinin 2b, and HFIAP-1. For all of these peptides (mostly similar), besides for Ascaphine-6_9286 APD_, we also identified membrane damaging properties. On the other, no antibacterial mechanism of action was reported for parents (of) P-10, or BF-CATH, a non-membrane damaging mechanism of action was reported for the YFGAP parents, and protein translation was reported for the parent of Pyrrhocoricin. We indicated different non-membrane damaging mechanisms, including protein translation inhibition for Pyrrhocoricin_7122 NCBI_, and delayed growth inhibition in M*e*^x^ for most of them. Nonetheless, a direct comparison between the data proposed by us and those for the parents in literature might be difficult, as small changes in the amino acids sequence might result in an alteration of the antimicrobial function^29^.

A limitation of the M*e*^x^ approach can be that the identified peptides are active intracellularly, but remain inactive when tested in MIC assays. For example, all 11 peptides derived from the parent PepG1 were among the most actives in M*e*^x^ (Figs. S8, S10, S12, S13), likely due to strong damage to the cytoplasmic membrane (Fig. 3a/b; Table 1). However, the most active candidate PepG1_11834 NCBI_, remained inactive at the tested concentration (60 µm) when added extracellularly. In fact, the PepG1 parent has shown very weak activity against Gram-negative bacteria (MIC of 25–100 µm) by others previously^30^. To overcome this limitation, uptake for such peptides could be enhanced by linking them to cell-penetrating peptides^31^.

Taken together, M*e*^x^ enables rapid discovery and classification of naturally-occurring and functionally diverse antimicrobial peptides. However, we argue that M*e*^x^ can also be used for de novo design or optimization of natural peptides by directed evolution approaches and that, the principal technology can eventually also be used for screening in drug-resistant (e.g. *Pseudomonas aeruginosa* or *Acinetobacter baumannii*). Ultimately, M*e*^x^ will hence allow paving the way towards the discovery of next-generation antibiotics.

## Methods

### Chemicals and reagents

Unless otherwise stated, all chemicals, reagents, and primers were obtained from Sigma Aldrich (Buchs, CH). Restriction enzymes and their buffers were obtained from New England Biolabs (Ipswich, USA). Synthetic genes were obtained from Integrated DNA Technologies (Leuven, BE) or Twist Bioscience (San Francisco, USA). Kits for plasmid isolation and DNA purification were obtained from Zymo Research (Irvine, USA). Peptides in either purified (> 90%) or crude format were obtained from Pepscan (Lelystad, NL). Sanger-sequencing was done at Microsynth (Balgach, CH).

### Bacterial strains and cultivations

Unless otherwise stated, all experiments were performed using *Escherichia coli* TOP10 (F^−^ *mcr*A Δ(*mrr*-*hsd*RMS-*mcr*BC) φ80*lac*ZΔM15 Δ*lac*X74 *rec*A1 *ara*D139 Δ(*ara-leu*)7697 *gal*U *gal*K λ^−^ *rps*L(Str^R^) *end*A1 *nup*G; Thermo Fisher Scientific, Waltham, USA). In this study, all cultivations were performed either in 14 ml polypropylene tubes (Greiner, Kremsmuenster, AT), filled with 5 ml of lysogeny broth (LB) medium (Difco, Becton Dickinson, Franklin Lakes, USA), or in 96-deep-well polypropylene plates (Greiner, Kremsmuenster, AT) filled with 500 µl of LB-medium. All samples were incubated at 37 °C with agitation on a shaker (Kuhner, Birsfelden, CH) operated at 200 r.p.m. and 25 mm amplitude. All media were supplemented with the appropriate antibiotic for plasmid maintenance (50 μg ml^−1^ kanamycin; 100 μg ml^−1^ carbenicillin) and 1% (w/v) d-glucose for repression of gene expression from catabolite-repression sensitive promoters such as P_BAD_. In the case of peptide expression experiments, cultures were incubated without d-glucose and 0.3% (w/v) of the inducer l-arabinose was used for induction. For all cultivations on solid medium, 15 mg ml^−1^ agar (Difco) was added to the broth, and incubation was performed without shaking in an incubator (Kuhner) at 37 °C. If not indicated differently, the optical densities (OD) of bacterial cultures were determined by measuring light scattering at 600 nm using a UV/VIS spectrophotometer (Eppendorf, Hamburg, DE).

### In silico generation of peptide library

We collected all peptide sequences (called “parents”) available on the APD in May 2017 (https://aps.unmc.edu/)^4^. These sequences were used as input queries to find sequence-similar peptide sequences in the NCBI non-redundant nucleotide collection (nr/nt), a collection that holds sequences from GenBank, European Molecular Biology Laboratory (EMBL), DNA Databank of Japan (DDBJ), and Reference Sequence database (RefSeq), as well as translated protein information from the protein database (PDB)^10^. By applying *tblastn*, 170,300 additional peptide sequences (called similars) were found^32^. Because we were limited to 12,412 different peptides with a maximum length of 42 amino acids (the chosen platform for the synthesis of the peptide-encoding oligonucleotides allowed 12,412 different sequences with a maximal length of 170 bases), we discarded similars with sequence similarity to the respective parent of less than 62.2%. The following parameters were used for the *tblastn* search: maximum sequences = 100; matrix = BLOSUM62; gap cost = 11.1; word size = 6; active low complexity filter; adjustment = conditional compositional score matrix adjustment.

### Sequence distance among parents and similar

To visualize sequence diversity among parents, we created a sequence-based phylogenetic tree. We performed pairwise global alignment of all parent sequences using the Needleman–Wunsch algorithm, as implemented in the R Bioconductor package ‘Biostrings’ (https://bioconductor.org/packages/release/bioc/html/Biostrings.html). The BLOSUM62 substitution matrix was used to compute the alignment scores, which were converted into pairwise distances following the method Scoredist^33^. Based on the pairwise distances between parents, we used hierarchical clustering with average linkage to compute a dendrogram of sequences reflecting their similarities. parents and their *tblastn*-derived similars were consolidated into groups, which were named after the parent from the APD (https://aps.unmc.edu/). In the sequence-based phylogenetic tree, each similar was stacked on top of its parent at the tip of the dendrogram. A similar may appear multiple times if it was found multiple times in the *tblastn* search using different parents.

### Peptide-encoding DNA architecture

The corresponding oligonucleotide sequences of the peptide library were synthesized using microarray technology supplied from CustomArray Inc. (now GeneString, Piscataway, USA). The chosen platform allowed 12,412 different oligonucleotides with a maximal length of 170 bases. A generic oligonucleotide design employing four functional units was created (Fig. S3): A coding unit, a filler unit, and two universal units for amplification. This process was automated for each sequence by using an in-house written script in R. The coding unit contained the reverse translation of the peptide amino acid sequence into a codon-optimized DNA for *E. coli*. We always chose the most abundant codon for each amino acid. In cases in which restriction sites had been introduced that could potentially interfere with subsequent manipulations, the crucial codon was replaced by the second most abundant one for this amino acid. The filler sequence was added to compensate for the various lengths of peptide genes (shortest coding sequence = 15 nucleotides, longest coding sequence = 126 nucleotides) and adjust the total of filler and coding unit to 129 nucleotides for all members of the library. To do so, we first added a UAA stop codon to the end of the coding sequence and then added downstream a semi-random sequence, ensuring a GC content of 40% for the filler sequence and limiting the number of identical nucleotides following each other to three. By adding this filler sequence we maximized sequence disparity at the DNA level (many coding sequences are homologs) thereby potentially increasing both synthesis and, later, sequencing quality. Two amplification units, of 23 and 18 bases, respectively, were appended upstream and downstream of the coding sequence and filler unit and contained the ribosomal binding site and restriction sites for the enzymes PstI and HindIII. Two amplify the peptide-encoding DNA, primer 1: CTGCACAAAGCTTACGTG, complementary to the upstream amplification unit, and primer 2: CACGTAAGCTTTGTGCAG, reverse complementary to the downstream amplification unit were used. The final 170 bases long oligonucleotide sequences as synthesized are listed by ID in File S2 (erroneous sequences were discarded).

### Peptide-encoding DNA cloning

The chemically synthesized and single-stranded oligonucleotides were separated from their array and we received them as a pool. This pool was aliquoted in 10 mM Tris–Cl, 1 mM EDTA, pH 8 and deep-frozen at − 80 °C. The pool was amplified by polymerase chain reaction (PCR) in a 50 µl reaction using 5 ng of the template and 10 µm HPLC-purified primer 1 and primer 2, complementary to the amplification sites, and 25 µl of Phusion High-Fidelity PCR Master Mix with HF buffer. The amplification was performed using 25 cycles of 98 °C for 15 s, 55 °C for 20 s, and 72 °C for 5 s. The now double-stranded peptide-encoding DNA sequences were purified using a DNA purification kit. DNA concentration was measured using a NanoDrop 2000 Spectrophotometer (Thermo Fisher Scientific) and 500 ng of the purified product was used for a restriction digest using enzymes HindIII-HF and PstI-HF in Cutsmart buffer. The digested product was again purified using a DNA purification kit and ligated to plasmid pBAD (Thermo Fisher Scientific) digested with the same enzymes^34^. This plasmid harbored the tightly controllable P_BAD_ promoter for peptide gene expression, a pBR322 replication of origin, and a resistance gene encoding for beta-lactamase. For ligation, pBAD was purified using a 1% agarose gel and a DNA gel recovery kit after digestion. Next, T4 ligase (800 units) was used to ligate 100 ng of cut pBAD vector and 10 ng peptide-encoding DNA sequences in T4 ligase buffer (molar ratio of 7:1 insert:vector). The ligation mix was incubated for 14 h at 16 °C. The ligation product was dialyzed in deionized water using filters (MilliporeSigma, Burlington, USA) and 1 µl of the mix was used to transform 20 µl of CloneCatcher Gold DH5G Electrocompetent *E. coli* (Genlantis, Burlington, USA) cells using electroporation. Recovered cells were plated and incubated overnight on LB agar plates supplemented with carbenicillin. Afterward, ~ 500,000 colonies were washed off the plates using LB medium, and the plasmids containing the peptide-encoding DNA sequences were extracted from 2.5 × 10^9^ cells using a plasmid isolation kit. An aliquot of 5 ng of these plasmids was used to transform *E. coli* TOP10 cells using the protocol from the transformation above. A total of 1,000,000 colonies were recovered from the plates after overnight incubation by washing with LB medium, the suspension was diluted to OD = 1 with LB-medium, glycerol was added to a final concentration of 20% (v/v), and aliquots of 500 million cells were stored at − 80 °C.

### Growth experiment

Three aliquots of *E. coli* TOP10 harboring the peptide-encoding DNA sequences on the pBAD plasmid were thawed and added to three 1 l baffled shake flasks containing 100 ml of LB medium + 100 μg ml^−1^ carbenicillin. The cultures were grown for roughly 7.5 h at 37 °C. When the OD reached 0.2, the cultures were supplemented with l-arabinose to a final concentration of 0.3% (w/v) to induce peptide expression. Cell samples were taken from each biological replicate at the point of induction and 1.5 h, 3 h, and 4.5 h post-induction. The plasmids were extracted from all samples using a plasmid isolation kit.

### NGS

For the generation of M*e*^x^ growth curves, peptide-encoding DNA sequences on plasmids, collected from the three replicates across four time points during the growth experiment, were sequenced by NGS. Additionally, the abundance of peptide-encoding DNA sequences in the original oligonucleotide pool and after transformation of the assay strain *E. coli* TOP10 was assessed by NGS as well. Peptide-encoding DNA sequences were amplified by primer 1 and primer 2 using 100 ng of plasmid and the PCR-amplification protocol mentioned before, but only for 10 cycles to avoid amplification bias. The amplification product was purified using an agarose gel. Single Index PentAdapters from Pentabase were used to prepare PCR-free libraries with the KAPA HyperPrep Kit (now Roche, Basel, CH) according to the manufacturer's specifications. Libraries were quantified using the qPCR KAPA Library Quantification Kit, pooled and sequenced PE 2 × 151 with an Illumina HiSeq 2500 using v4 SBS chemistry. Roughly 10% genomic PhiX library as spike-in to increase sequence diversity. Basecalling was done with bcl2fastq v2.20.0.422. The resulting fastq files were processed using in-house software written in R and C. This software aligns each sequence to our reference table of 12,412 sequences linking peptide-encoding DNA sequences and peptide sequence, identifies mismatches and sequencing errors, and counts how often each peptide-encoding DNA sequence was sequenced in each sample. NGS read counts for each sequence analyzed in M*e*^x^ were listed with a unique identifier (ID) in File S2.

### Generation of M*e*^x^ growth curves

We used the standard workflow of DESeq2 (NGS read count normalization, dispersion estimates, and Wald’s tests) to analyze NGS read counts^35^. Only sequences that passed independent filtering were included in further analyses (= 10,633). To draw growth curves for each peptide-expressing strain, we calculated the log2-fold changes of NGS read counts (listed for each ID in File S2) between the time of induction and all other time points (1.5 h, 3.0 h, and 4.5 h post-induction). A Bayesian shrinkage estimator was employed to shrink the log2 fold-change for each ID (lfcShrinks_ID_) between all time points using the R/Bioconductor package ‘apeglm’^36^. To draw the M*e*^x^ growth curves, we calculated a strain-specific OD_ID_ at each time point according to Eq. (1). OD values at the specific time points were averaged values from all three biological replicates (Fig. S5). The OD_ID_ (0 h) for each peptide-expressing strain was set to 0.2 at the time of induction as lfcShrink_ID_ (0 h) = 0 and OD = 0.2. This enabled us to compare peptide-expressing strains of different abundancies (see Fig. S6). OD_ID_ values can be interpreted as the OD values that would have been measured when incubating the respective strain individually in the same experiment, i.e. in this case in LB medium in a 100 ml shake flasks.

1 $${\text{OD}}_{{{\text{ID}}}} \left({\text{t}} \right)={\text{OD}} \left({\text{t}} \right) \times 2^{{{\text{lfcShrinkID}} \left({\text{t}} \right)}}$$

To find M*e*^x^-active peptides, we also performed a one-sided Wald’s test, with the alternative hypothesis that the expression of a given peptide leads to a reduced OD_ID_ 1.5 h and 4.5 h post-induction. We rejected the null hypothesis at significance level alpha = 0.05. Peptides with a *p* < 0.05 (after adjustment for multiple testing using the Benjamini–Hochberg method) after 4.5 h are considered M*e*^x^-active peptides. Peptides with *p* < 0.05 after 1.5 h do significantly inhibit growth already after 1.5 h. All values and results are reported in File S1.

### Monoseptic growth experiments

Taking the OD_ID_ (4.5 h) of each peptide-expressing strain, we could rank all peptides by their growth inhibitory effect. We selected 110 peptides (Ranks 1–50, 100–119, 1000–1019, and 10,000–10,019) and then generated an identical copy of the strain previously used in M*e*^x^ for its expression. First, the corresponding peptide-encoding DNA-sequences were synthesized as gene fragments. An aliquot of 400 ng of each gene fragment was directly used for a restriction digest using enzymes HindIII-HF and Pst-HF in Cutsmart buffer. The product was purified using a DNA purification kit. Next, T4 ligase (800 units) was used to ligate 50 ng of identically digested pBAD vector and 10 ng of purified gene fragment in T4 ligase buffer for 14 h at 16 °C. The ligation product was purified using a DNA purification kit. An aliquot of 5 µl of the purified ligation product was then used to transform chemically competent *E. coli* TOP10 cells. From the resulting colonies, we isolated one strain, sequence-verified the correct assembly of the expression plasmid, and stored it after overnight growth in glycerol at − 80 °C. For the growth experiment, we first re-isolated single colonies on solid media and then picked three clones, incubated them separately overnight, and inoculated them into 200 µl fresh LB medium containing 0.3% (w/v) l-arabinose to a final OD of 0.01 into 96-well microtiter plates (Greiner). Growth was recorded by measuring OD in a Tecan Infinite 200 PRO (Tecan, Männedorf, CH) for 4.5 h (37 °C, 1.5 mm orbital shaking).

### Enrichment analyses

We used Fisher’s exact test to assess the over- or underrepresentation of M*e*^x^-actives in various groups. This amounts to a hypergeometric test to assess the significance of drawing *n* active peptides in a group of *k*, from a population of size *N* containing *K* active peptides. We rejected the null hypothesis at significance level alpha = 0.05. Groups with a *p* < 0.05 had a significantly different representation of active peptides compared with the overall population. When adjusting for multiple testing, we used the Benjamini–Hochberg method.

### Peptide classifications

The physicochemical parameters of the peptides were calculated at pH 7 using the R package ‘Peptides’ (https://cran.r-project.org/package=Peptides). For charge, we used the method by Lehninger^37^. For hydrophobicity (or GRAVY index), we used the calculations by KyteDoolittle^38^. The information for each parent such as the name, chemical modification, activity, 3D-structure, was extracted from the APD website (https://aps.unmc.edu/) using an in-house R script. The information on the species from which a specific peptide sequence originated, was extracted from the *tblastn* search and the APD website. The entire taxonomic classifications (kingdom, phylum, class) for each species were extracted, if available, from the Global Biodiversity Information Facility Data Portal (https://gbif.org) using the R package ‘taxize’ (https://cran.r-project.org/package=taxize). The results are summarized in File S1.

### Membrane damage assay using intracellularly synthesized peptides

We selected the peptide-expressing strains of rank 1–50 in M*e*^x^ that we had previously constructed for the monoseptic growth assay. Additionally, we selected the strain expressing the inactive control peptide HNP-1_3425 APD_, a peptide known to be inactive if expressed in *E. coli*^8^. Each strain was re-isolated on solid media from frozen stock and incubated overnight. Then, two colonies were picked and incubated overnight in 96-deep-well polypropylene plates. These cultures were used to inoculate fresh media containing 0.3% (w/v) l-arabinose to a final OD of 0.01 into 96-well microtiter plates. The plates were then incubated on for 4.5 h (37 °C, 1.5 mm orbital shaking). After 4.5 h, an aliquot of 50 µl of cell suspension a Tecan Infinite 200 PRO plate reader was added to 150 µl of phosphate-buffered saline into a fresh 96-well microtiter plate. Propidium iodide (PI) was added to a final concentration of 1 µg ml^−1^. PI is a DNA-intercalating dye that cannot pass an intact cytoplasmic membrane^39^. For each sample, PI fluorescence (λ_Ex_ = 579 nm/λ_Em_ = 616 nm) of ~ 10,000 cells were analyzed using a flow cytometer LSR Fortessa (BD Biosciences, Allschwil, CH). To determine the membrane damaging properties of each of the expressed peptides, we calculated the fraction of cells in percent for which a PI uptake was measured using the software FlowJo V10 (BD Biosciences).

### Stress response assay using intracellularly synthesized peptides

We selected peptide-expressing strains of rank 1–50, previously generated for the monoseptic growth assay. Additionally, we selected the strain expressing the inactive control peptide HNP-1_3425 APD_. Moreover, two plasmids (cloning vector: puA66) containing either the promoter of the gene for recombinase A (P_recA_) or for the gene for cold shock protein A (P_cspA_) were purified from the *E. coli* Alon collection^15^. Both plasmids contained a transcriptional fusion of their promoter with a downstream gene for green fluorescent protein (*gfp*), an additional kanamycin resistance cassette, and a pSC101 origin of replication. We transformed each of the 51 peptide-expressing *E.* *coli* strains with each of the two plasmids to generate 102 different strains and incubated them overnight on solid media. Then, three colonies were picked and incubated overnight. These cultures were used to inoculate fresh media containing 0.3% (w/v) l-arabinose to a final OD of 0.05 into 96-well microtiter plates. We recorded OD and GFP expression (λ_Ex_ 488 nm/λ_Em_ 530 nm) after 1.5 h and 4.5 h using a Tecan Infinite 200 PRO (37 °C, 1.5 mm orbital shaking). For each strain, we calculated the specific fluorescence change between the two time points [GFP/OD (4.5 h)–GFP/OD (1.5 h)]. Statistical significance was calculated by one-sided *t* tests, adjusted for multiple testing by Benjamini–Hochberg, using the signal of HNP-1_3425 APD_ as null distribution. We rejected the null hypothesis at significance level alpha = 0.05.

### Purification of chemically synthesized peptides

Peptides were obtained from Pepscan (Lelystad, NL) in > 90% purity or in crude format and subsequently purified to > 90% purity in-house. For the latter, crude peptides were dissolved in 5 ml DMSO and 15 ml 0.1% aqueous trifluoroacetic acid, TFA. HPLC-purification of the dissolved crude peptides was performed on an ӒKTAexplorer chromatography system (GE Heathcare, SE). The entire peptide sample was loaded onto a RP C18 column (PRONTOSIL 120 C18 10 μm, 250 × 20 mm, 50 × 20 mm precolumn, Bischoff, Leonberg, DE), heated to 30 °C and operated at a flow rate of 10 ml min^−1^ using 0.1% aqueous TFA as solvent A and acetonitrile supplemented with 0.1% TFA as solvent B. The ratios of A to B were adapted for each peptide and typical values are given below. The column was equilibrated with the peptide-specific mixture of solvent A and solvent B (0–20%) prior to injection. After injection and an initial wash step of 6 min a gradient was imposed with the same mixture, and then a gradient was applied, in the course of which the amount of solvent B was increased to 50–90% in 40 min. The column was washed with 95% solvent B for 8 min and equilibrated with the specific solvent A/solvent B mixture for the next run for 13 min. Peptide elution was monitored spectrophotometrically at 205 nm, and generally the main peptide peak was collected. The sample was frozen at − 80 °C for > 2 h and lyophilized (approx. 18 h) using a freeze-dryer (Alpha 2–4 LDplus, Christ, DE), connected to a vacuum pump (RC6, Vacuubrand, DE). The lyophilized peptides were dissolved in 1 ml DMSO and stored at − 20 °C. The concentration of the peptide stocks was determined via HPLC using an Agilent 1200 series HPLC system. Each peptide stock was analyzed as a 1:100 dilution in water. An aliquot of 10 μl of the peptide stock was injected onto an RP‐C18 column (ReproSil‐Pur Basic C18, 50 × 3 mm, Dr. Maisch, Germany) operated with water supplemented with 0.1% TFA as solvent A and acetonitrile supplemented with 0.1% TFA as solvent B. Separation was performed using the same concentration profile previously used for purification. The concentration was measured using the integrated peak area at 205 nm and then calculated using peptide-specific absorption properties^40,41^.

### Measurement of the MIC using chemically synthesized peptides

On the same day at which MIC assays were executed, purified peptides were thawed and the concentration was determined by HPLC as described before. *E. coli* TOP10 cells were grown in Mueller Hinton Broth (MHB) or diluted MHB (25% of the original strength) overnight to stationary phase. Diluted MHB has been frequently used to assay antimicrobial peptides^42^. The cultures were then supplemented with 20% glycerol, aliquoted, and frozen at − 80 °C. For MIC measurements, a frozen stock of the cells was thawed, resuspended in MHB or 25% MHB to adjust to a density of 5 × 10^5^ cells ml^−1^ in the experiment, and distributed to microtiter plate wells by an automated liquid handling system (Hamilton, Bonaduz, CH). Then the peptides were added by the liquid handling system in twofold dilutions using minimum of 100 µg ml^−1^ as the highest concentration. MICs were determined as broth microdilution assay in 384-well flat bottom polypropylene plates (Falcon 96-Well Flat-Bottom Microplate) adapted from the protocol of Wiegand et al.^43^. The plates were sealed airtight and incubated for 18 h without shaking at 37 °C before reading the OD using a Tecan Infinite 200 PRO plate reader. The MIC value corresponded to the concentration at which no growth of the bacterial strain was observed (< 5% of the OD value of the growth control). MIC experiments were performed at least in triplicate.

### Membrane damage assay using chemically synthesized peptides

To measure extracellular membrane damage, *E. coli* TOP10 [pSEVA271-GFP] and the peptide dilutions were prepared as described for the MIC measurements (using 100% MHB as medium) but covering a concentration range of 16 × MIC to MIC/16 in twofold dilutions steps with a final assay volume of 200 µl. The bacterial strain suspension was furthermore supplemented with a final concentration of 1 µg ml^−1^ propidium iodide just before pipetting the assay. After 1 h incubation at room temperature membrane damage (= release of intracellularly expressed GFP and/or uptake of extracellularly added PI) was assessed by flow cytometry using a Fortessa Analyzer (BD Biosciences; 488 nm laser with 530/30 nm bandpass filter and 579 nm laser with 610/20 nm bandpass filter). The fractions of PI-positive and GFP-positive were determined with the same gate for all populations using the FlowJo V10 software (BD Biosciences). The extracellular membrane integrity assay was performed in biological duplicates analyzing at least 10,000 cells in each experiment.

### Hemolysis assay using chemically synthesized peptides

Two samples of human blood were obtained from a blood bank (Blutspendezentrum SRK at the University Hospital Basel). The samples were pooled and erythrocytes were isolated by repeated centrifugation at 500×*g* for 10 min, removal of the blood plasma and resuspending the remaining cells in an equal volume of DPBS. Following last resuspension, erythrocytes were diluted 1:50 in DPBS. For the hemolysis assay, a log2 serial dilution of each peptide was prepared as described for the MIC but using DPBS and a 96-well plate (U-bottom, PP, 650201, Greiner) with a final volume of 200 µl. As lysis control, 2.5% Triton-X100 in DPBS was used in well 10, well 11 served as non-treated control (no peptide added), and well 12 as blank. To each well of the dilution plate, 100 μl of the red blood cells suspension was added. The plate was incubated for 1 h at 37 °C. After the incubation, the plate was centrifuged at 500×*g* for 10 min and 100 µl of the supernatant was transferred to a clean 96-well plate (F-bottom, PS, 655101, Greiner). The absorbance was measured at 540 nm using an Infinite M1000 PRO plate reader (Tecan) and corrected by the measurements from the untreated wells. The lysis of each peptide concentration was expressed relative to the lysis control (set as 100% lysis). The hemolysis assay was performed in triplicate.

## Supplementary Information

Supplementary Information 1.

Supplementary Information 2.

Supplementary Information 3.
