# Supplementary material for: Discovery of antimicrobials by massively parallelized growth assays (Mex)
Source: Sci Rep. 2022 Mar 8;12:4097. doi: 10.1038/s41598-022-07755-7 (PMC8904554; doi:10.1038/s41598-022-07755-7)
Supplement: Supplementary file 3 — Supplementary Information 3. [file 41598_2022_7755_MOESM3_ESM.docx]

Supplementary Information

**Discovery of antimicrobials by massively parallelized growth assays (M*e*^x^)**

Philipp Koch^1^, Steven Schmitt^1^, Mathias Cardner^2,3^, Niko Beerenwinkel^2,3^, Sven Panke^1^, Martin Held^1*^

All relevant information for each peptide analyzed in M*e*^x^ (amino acid sequences, physiochemical properties, taxonomical information, p-values, OD_ID_-values, link to APD) is provided in **File S1**. NGS read counts and all peptide-encoding DNA sequences are provided in **File S2**. The computational workflow to reproduce the analysis of **File S1** and generate growth curves of each peptide expressing-strain is available on GitHub (https://github.com/derpkoch/MeX). NGS data are available at the NCBI Sequence Read Archive (SRA) under accession number PRJNA686958. Additional data that support the findings of this study are available from the corresponding author upon reasonable request.


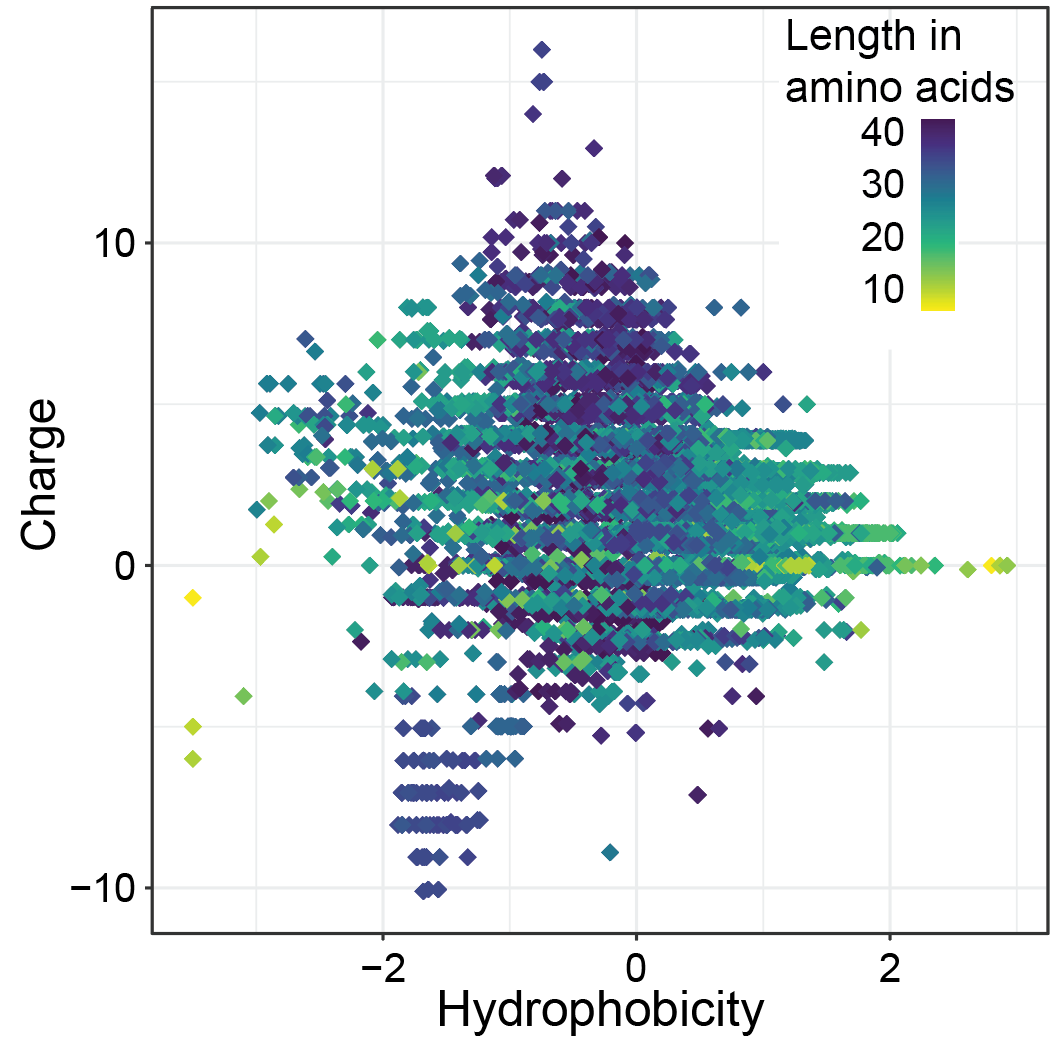


**Figure S1 | Predicted physiochemical properties of peptides in the library.** All peptides of the library are plotted according to their charge and hydrophobicity at pH 7 and colored by their length. Mean charge = +2.3; mean hydrophobicity (GRAVY scale) = 0.0; mean length = 27 amino acids.


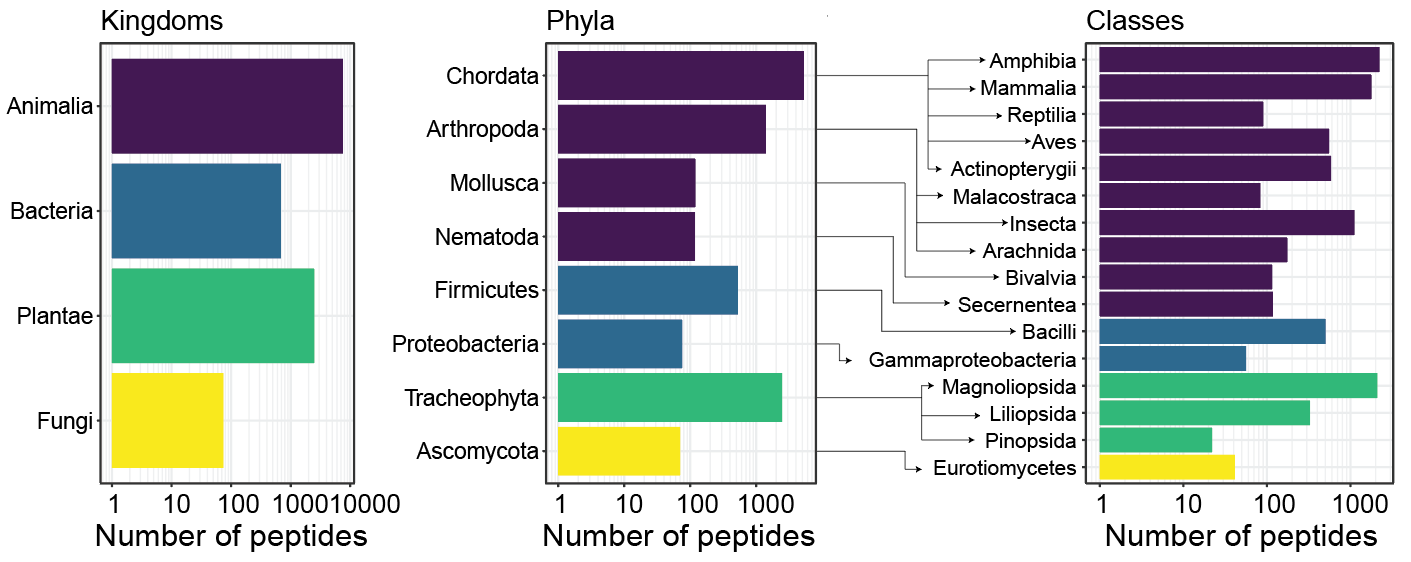


**Figure S2 |Taxonomical classification of the peptide library.** All peptides of the library are grouped by their taxa in rank kingdom, phylum, and class of the host from which their sequences had been derived. Only groups comprised of at least 20 peptides are displayed. Phyla and classes are colored by their kingdom (left).


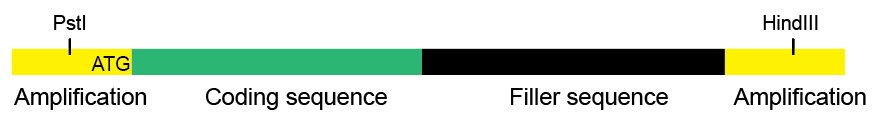


**Figure S3 |** Sequence architecture of peptide-encoding DNA sequences as used for synthesis. Each DNA sequence contains a peptide coding sequence (green), a unique filler sequence (black) used for standardizing sequence length to 170 nucleotides, and two universal amplification sites (yellow) used for both, cloning and amplification. The coding sequences are generated by reverse translation of the respective peptide amino acid sequence followed by codon optimization for expression in *E. coli*. Amplification sites at the 5’ and 3’ end are the same for all inserts and contain restriction sites for subsequent integration into the multiple cloning site of the expression plasmid.


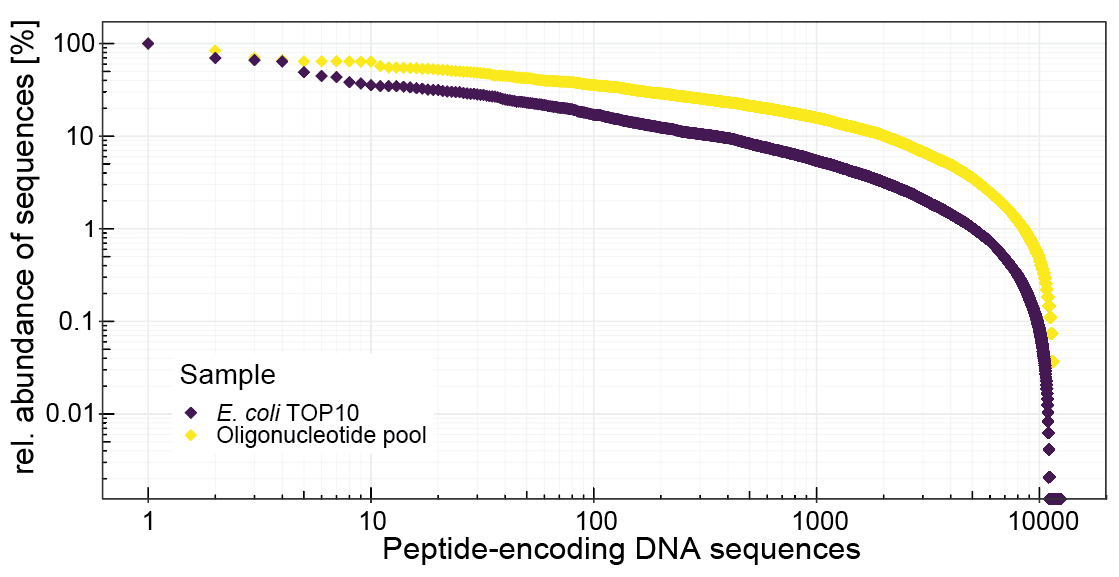


**Figure S4 |** Relative abundance of peptide-encoding DNA sequences before and after cloning. Peptide-encoding DNA sequences in the original synthesized oligonucleotide pool (yellow) and after insertion into a plasmid and transformation of *E. coli* TOP10, as used for the growth experiment (purple) are counted by NGS. All counts are relative to the most abundant peptide-encoding sequence Chensinin-1CEb_2720 NCBI_, which appears 2,720 times (oligonucleotide pool) and 5,466 times (growth experiment), respectively.


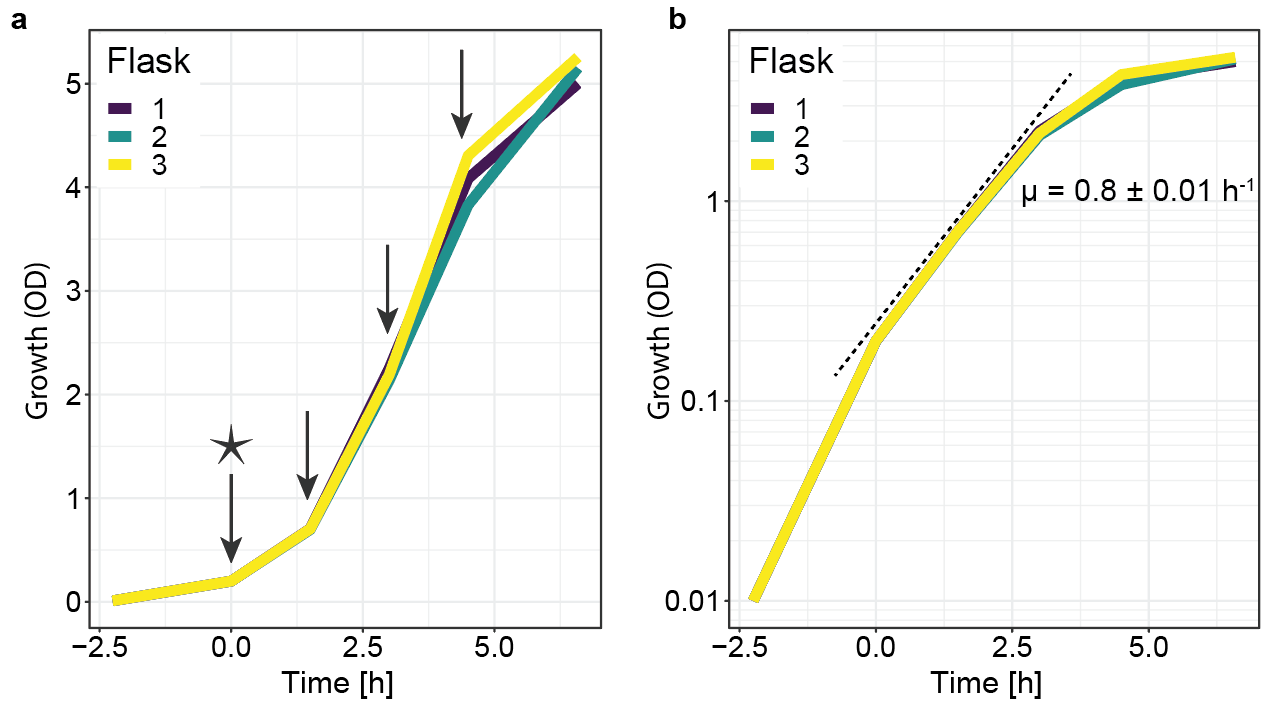


**Figure S5 | Growth of *E. coli* TOP10 expressing the peptide-encoded DNA library**. **a)** Optical density at 600 nm (OD) is recorded over 8 h. Three 1 liter shake flasks containing 100 ml of LB-medium each are inoculated with 500 million cells of *E. coli* TOP10 carrying the peptide-encoded DNA library at -2.5 h (time reported relative to the time of induction). Peptides are expressed after 4 generations (0.0 h; OD~0.2) by adding l-arabinose (asterisk). Cell samples for NGS are isolated from each replicate at the time of induction, and 1.5 h, 3.0 h, and 4.5 h post-induction (arrows). **b)** Log10 transformed data of (a) for calculation of the specific growth rate (µ).


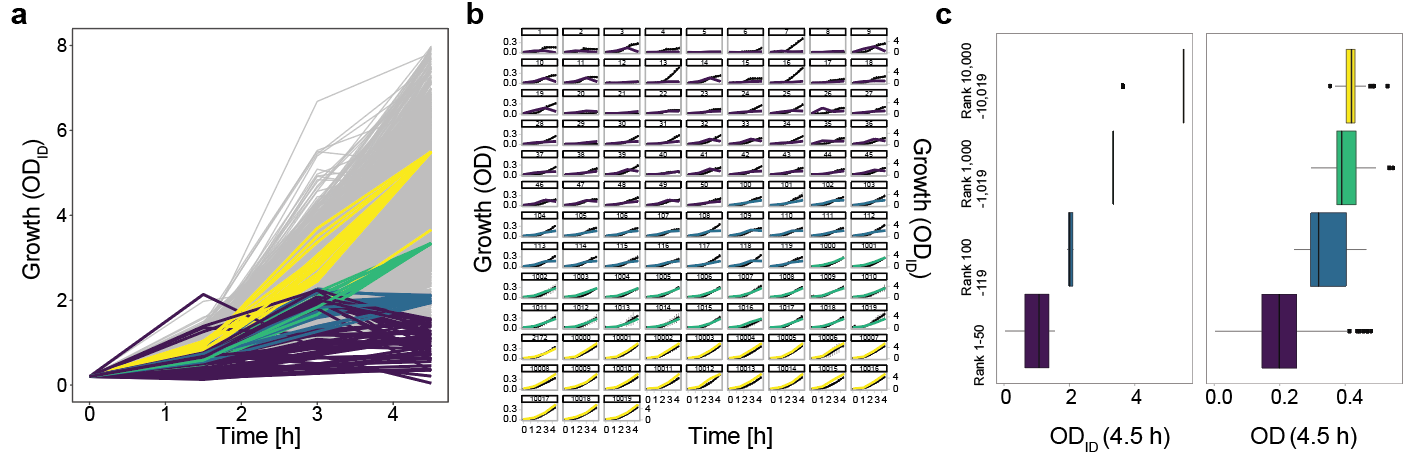


**Figure S6 |** Growth of peptide-expressing *E. coli* strains in M*e*^x^ and in monoseptic cultures. **a)** All peptide-expressing strains are ranked by their growth inhibition after 4.5 h (= ranked by their OD_ID_ (4.5 h)) and selected representatives were then subdivided into four groups: purple = rank 1-50 (average OD_ID_ (4.5 h) = 1.0); blue = rank 100-119 (average OD_ID_ (4.5 h) = 2.0); green = rank 1,000-1,019 (average OD_ID_ (4.5 h) = 3.3); and yellow = rank 10,000-10,019 (average OD_ID_ (4.5 h) = 5.4) and HNP-_13425 APD_ (negative control, rank 2172, OD_ID_ (4.5 h) = 3.7 ). **b)** Overlay of growth curves recorded by M*e*^x^ (colored lines; average of n=3) and during monoseptic growth of the same strains in microtiter plates (black lines; n=3, error bars = 4σ). Numbers on top of each recording correspond to M*e*^x^-derived activity rank. **c)** Boxplot of OD_ID_(4.5 h) and OD (4.5h) of the peptide-expressing strains in the different subgroups identified in (a). Note the different scales on the x-axis. Rank 1-50: average OD (4.5h) = 0.2. Rank 100-110: average OD (4.5h) = 0.35. Rank 1,000-1,019: average OD (4.5h) = 0.4. Rank 10,000-10,019: average OD (4.5h) = 0.42.


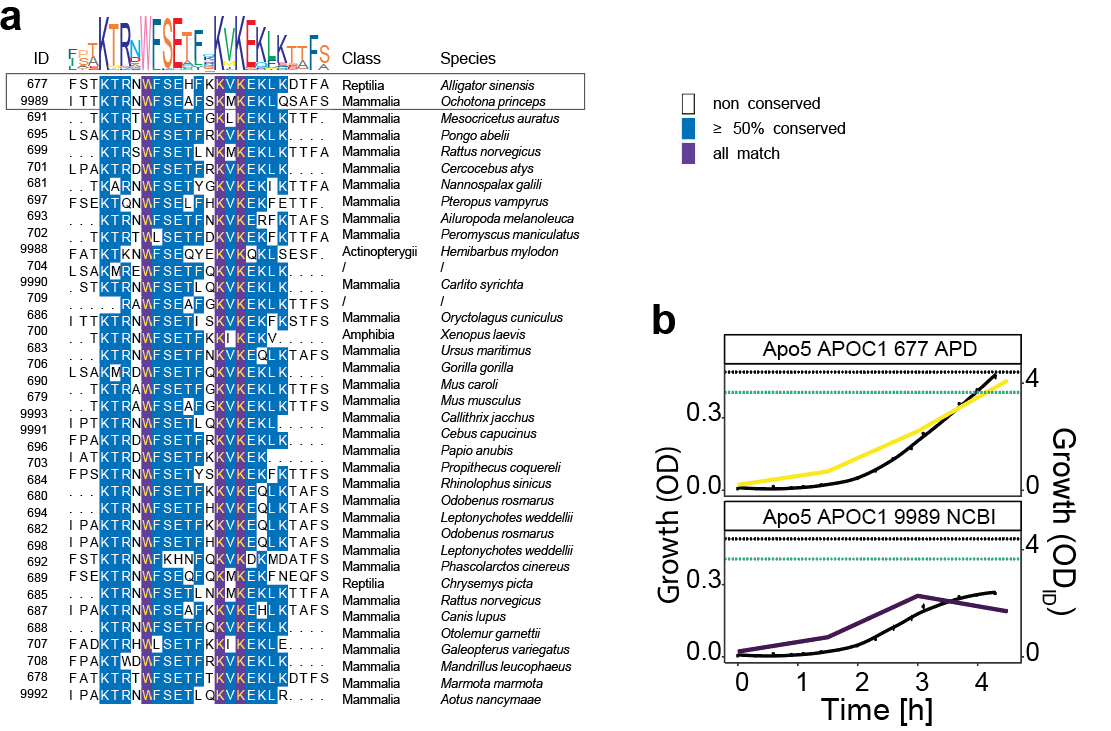

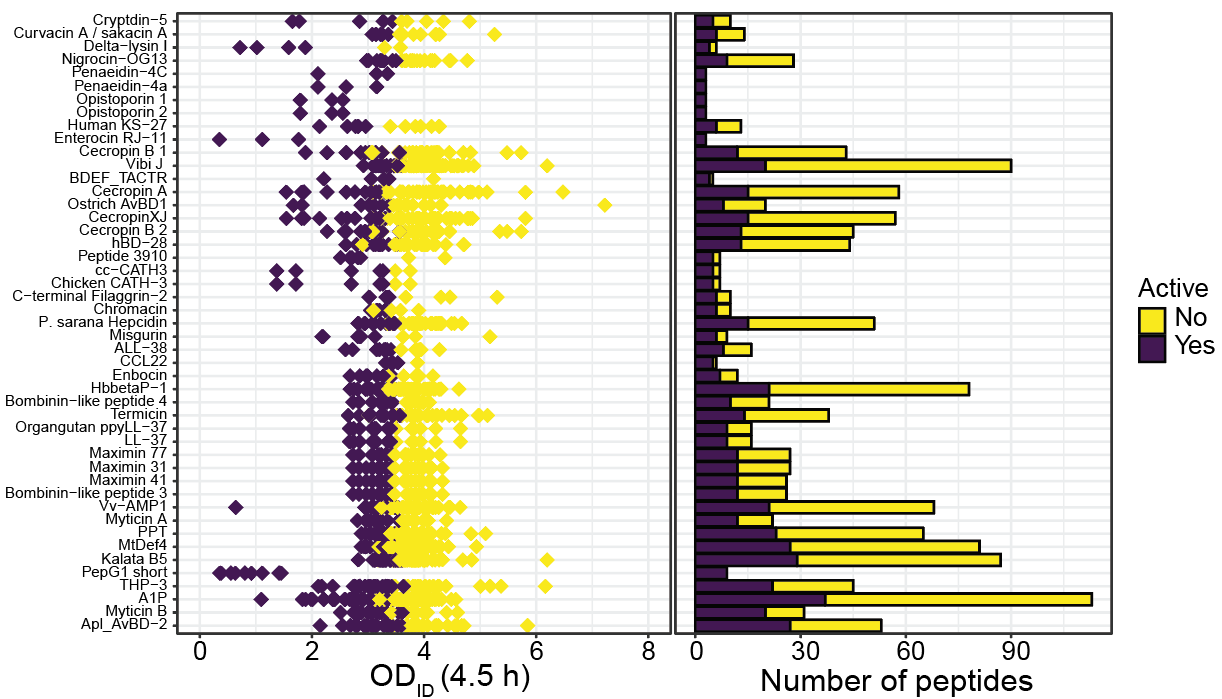


**Figure S7 | Analysis of Apo5 APOC1-derived similars. a)** Amino acid sequence alignment of all 36 similars of the Apo5 APOC1_677 APD_ parent. The inactive parent, derived from Chinese alligator (*Alligator sinensis*), and the only M*e*^x^-active similar (Apo5 APOC1_9989 NCBI_) derived from American pika (*Ochotona princeps*) differ by nine amino acids. The top shows the consensus sequence plot. **b)** Overlay of growth curves recorded by M*e*^x^ (colored line, an average of n=3) and via monoseptic growth in microtiter plates (black line; n=3, error bars = 2σ), of *E. coli* TOP10 cells expressing Apo5 APOC1_677 APD_ and Apo5 APOC1_9989 NCBI_. Horizontal dashed lines, in black (OD) or colored in green (OD_ID_), show final values measured 4.5 h post-induction of a strain synthesizing the inactive control peptide HNP-1_3425 APD_ (obtained from results displayed in Supplementary Fig. 14).

**Figure S8 | Overrepresentation of active similars derived from 47 parents.** For 47 parents (names on the left), M*e*^x^-actives were significantly overrepresented among the similars identified in the similarity search (Fisher’s exact test, adj. *p*<0.05). The OD_ID_ (4.5 h) values for the individual peptide-expressing strains within a group of parents and similars are shown as dots (left) and the total number of active and non-active peptides for each of the 47 parents as bars (right).


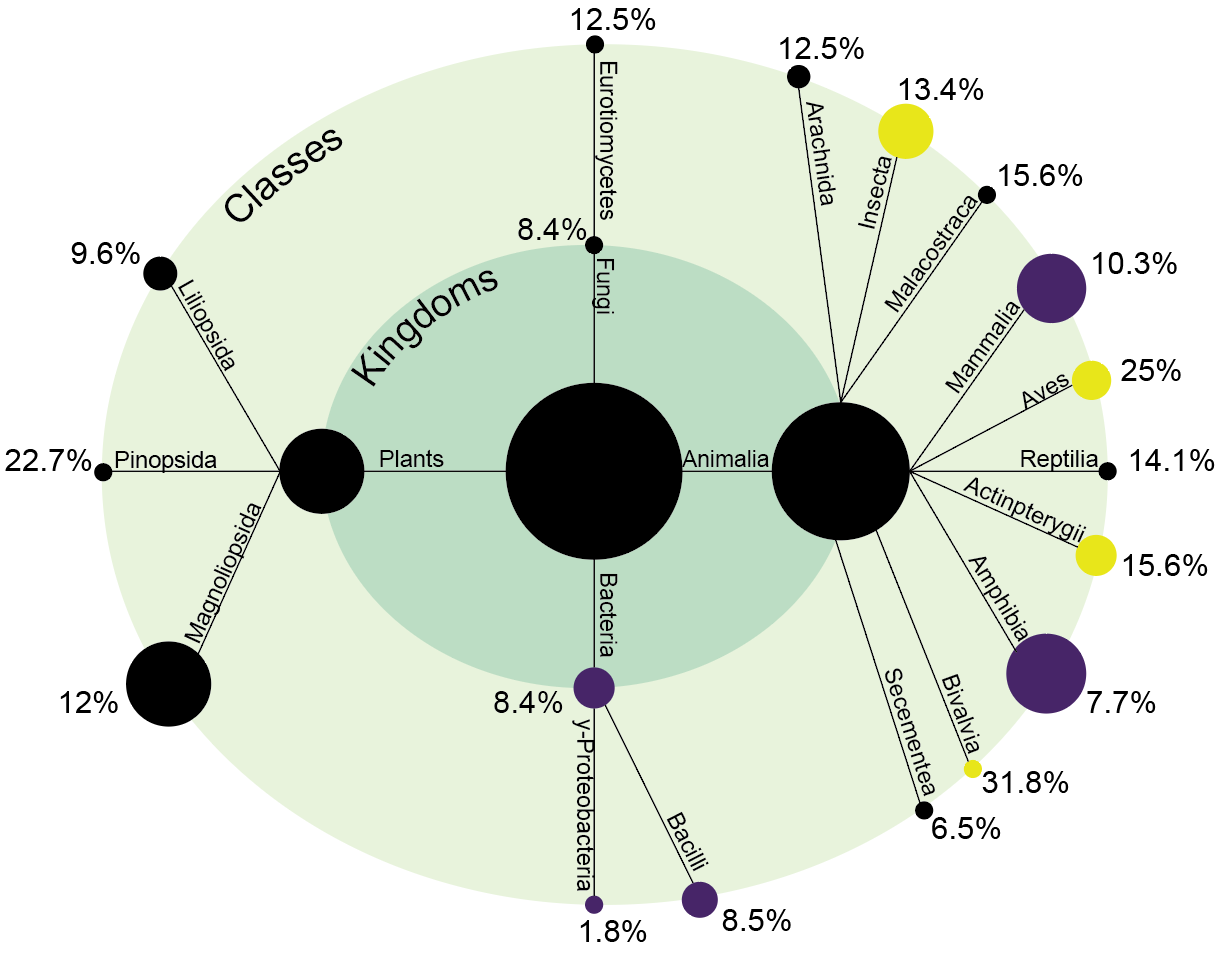


**Figure S9 | Taxa-specific M*e*^x^-activity in rank kingdom and class.** All peptides tested by M*e*^x^ are clustered taxonomically according to the host from which they were derived. The percentage of M*e*^x^-actives in the cluster is written next to (or inside) each circle. Compared to the 11.6% M*e*^x^-actives in the entire library, taxa in which M*e*^x^-actives are over- (yellow) or underrepresented (purple) are highlighted (*p*<0.05). Only clusters with more than 20 peptides are displayed. The circle area is representative of the total number of peptides within each cluster.


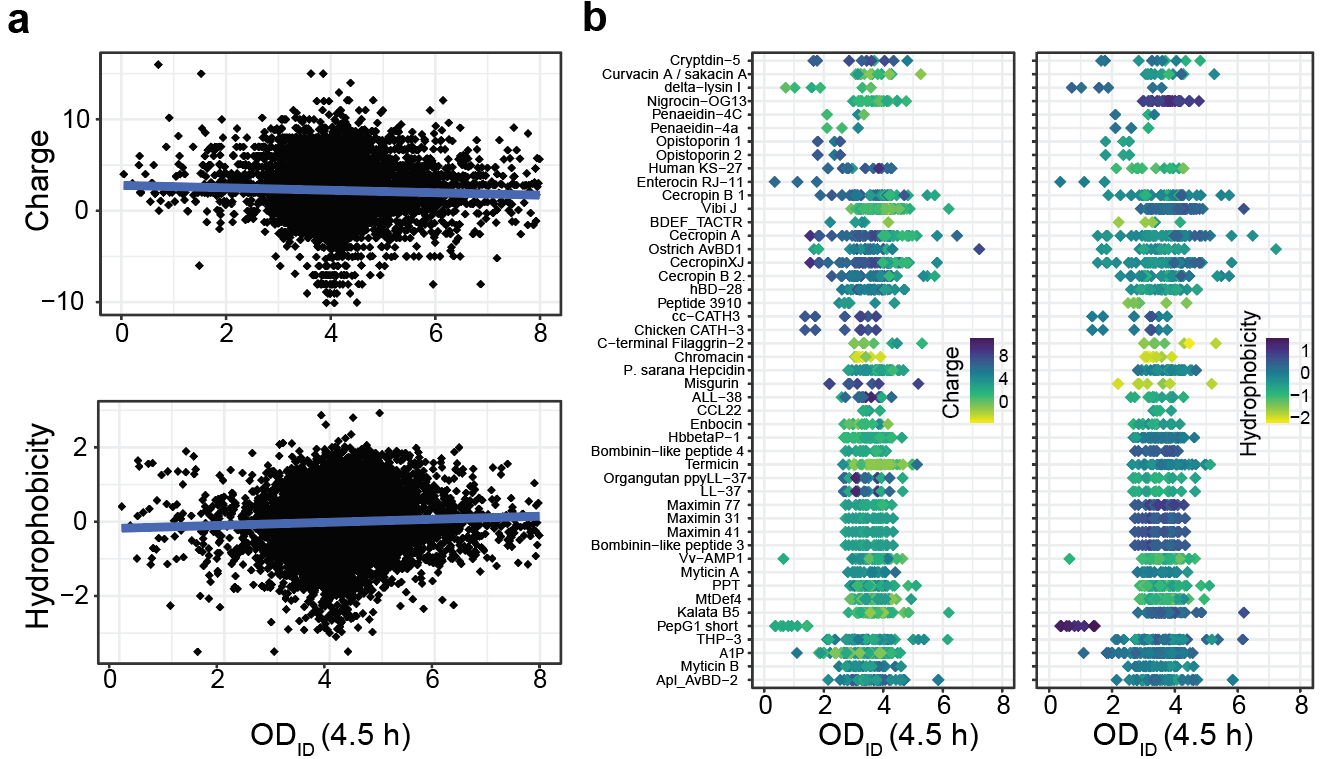


**Figure S10 | Influence of the physicochemical properties of peptides on M*e*^x^-activity. a)** Charge (top) and hydrophobicity (bottom) of each peptide assayed by M*e*^x^ are plotted against OD_ID_ (4.5h). Linear fits (both *p*<0.01, R^2^<0.001, 10,661 DF) are displayed for the entire peptide library (blue line) **b)** Charge (left) and hydrophobicity (right) are displayed for the 47 groups of parents and their similars containing an overrepresentation of M*e*^x^-positives (Fisher’s exact test, adj. *p*<0.05) as shown in Supplementary Fig. 9.


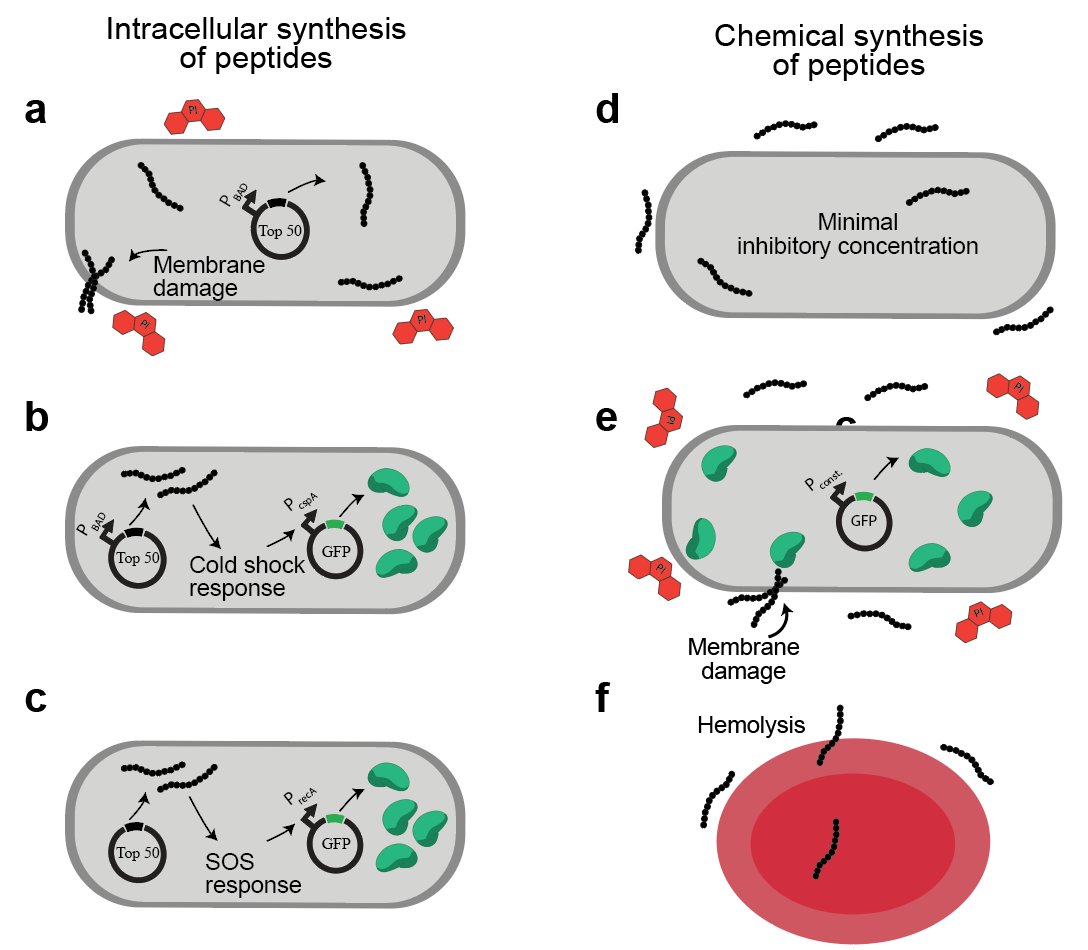


**Figure S11 | Assays for activity assessment of antimicrobial peptides. (a-c)** Intracellular synthesis of peptides. The 50 most active peptides as indicated by M*e*^x^ (ranks 1-50; by OD_ID_ (4.5 h)) are expressed intracellularly in *E. coli* TOP10 with or without a plasmid for the expression of green fluorescent protein (GFP) from a promoter, whose activity has been linked it a specific cellular stress response. **a)** Analysis of membrane damage by quantification of propidium iodide (PI) uptake. **b&c)** Quantification of GFP indicating interference with intracellular targets by eliciting an SOS stress response indicative of DNA damage (readout via P*_recA_* (promoter of *E. coli*’s *recA* gene)) or cold shock response indicative of translation inhibition (readout via P*_cspA_* (promoter *E. coli*’s *cspA* gene)). **(d-f)** The 20 most M*e*^x^-active peptides are synthesized chemically and purified. **d)** The minimal inhibitory concentration (MIC) is determined by adding chemically synthesized peptides to cultures of *E. coli* TOP10 and other pathogens (**Table 1** & **Table S1**). **e)** Membrane damage is determined by quantifying uptake of PI and the release of intracellularly expressed GFP after addition of the peptides to cultures of *E. coli* TOP10. **f)** Cellular toxicity is determined by measuring the degree to which human erythrocytes are lysed in the presence of chemically synthesized peptides.


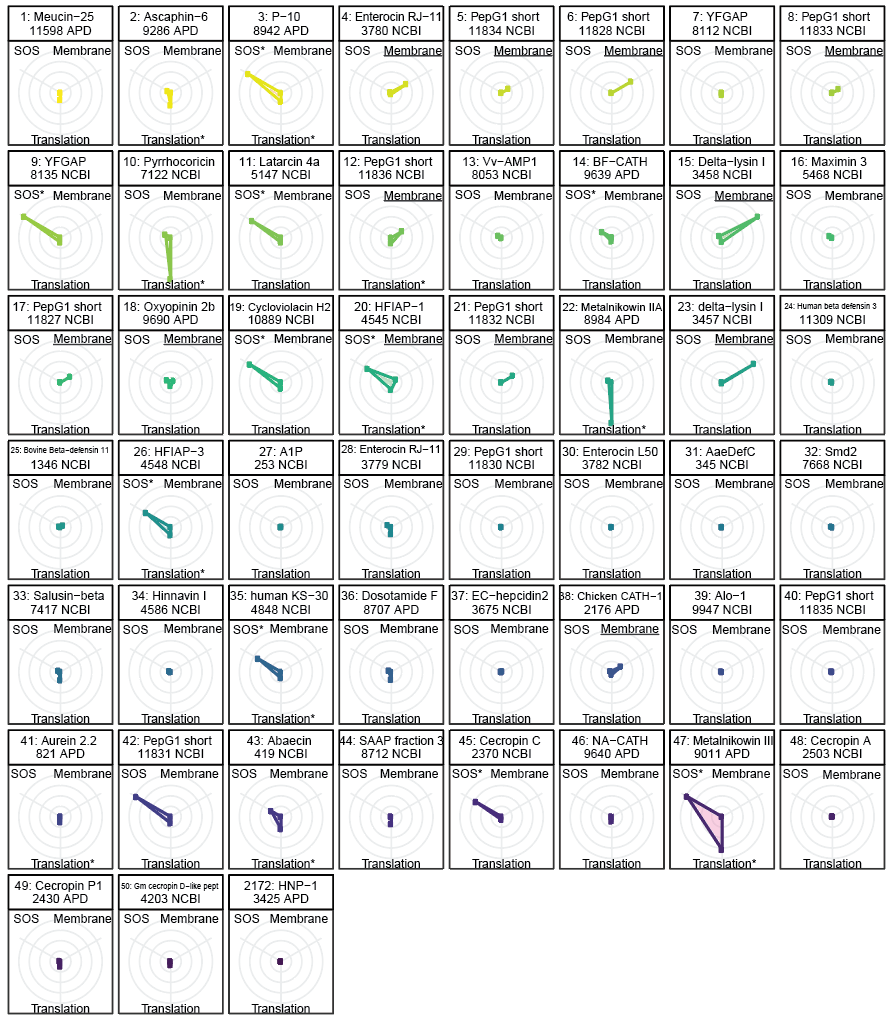


**Figure S12 | Characterization of the 50 most active peptides in M*e*^x^ as addition to Fig. 2.** Potential mechanisms of action. Each radar plot shows the mean SOS-response (DNA; activation of the *recA* promoter; n=3), translation inhibition (Translation; activation of the *cspA* promoter; n=3) and membrane-damage (Membrane; PI stained cells in percent; n=2) obtained after peptide expression in *E. coli* TOP10. Only the maximum and minimum values are reported in digits. The center represents values measured for the negative control peptide HNP-1_3425 APD_. Lower values are scaled to the center. Membrane damage is attributed if more than 10% of cells were PI-positive (underlined). For SOS and Translation, signals are reported relative to the signal obtained for the inactive control peptide HNP-1_3425 APD_. A significant increase (one-sided t-test, adj. *p*<0.05) compared to the inactive control is indicated by an asterisk (*).


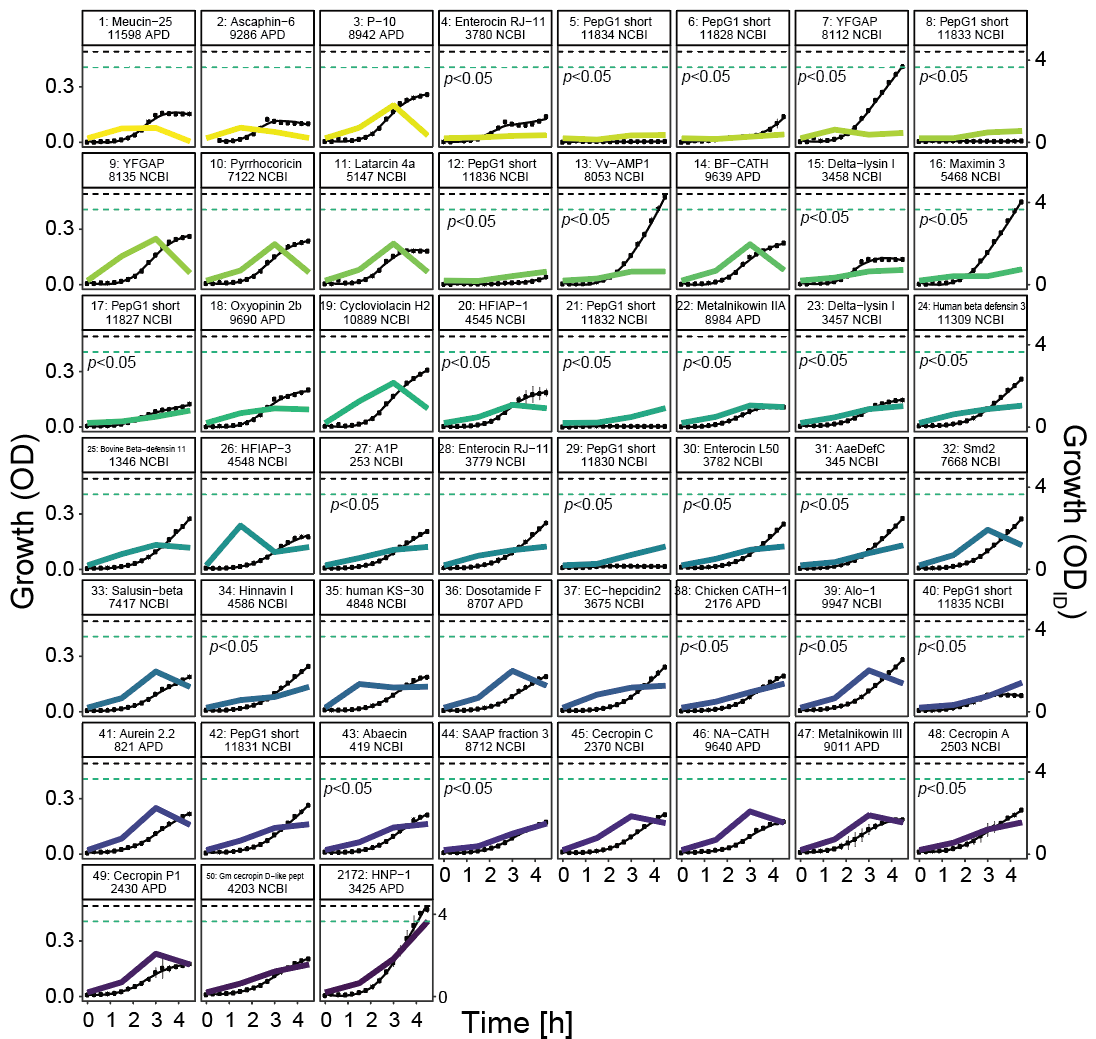


**Figure S13 | Characterization of the 50 most active peptides in M*e*^x^ as addition to Fig. 2.** Growth curves of the 50 most active peptides. Colored lines are M*e*^x^-recorded growth curves (average of n=3) determined via OD_ID_ approximation (header: ‘rank: parent name’). Black lines are growth curves (n=3, error bars: 4σ) determined via OD measurement in microtiter plates of individually grown strains. Horizontal dashed lines, in black (OD) or colored in green (OD_ID_), show final values measured 4.5 h post-induction of a strain synthesizing the inactive control peptide HNP-1_3425 APD_. In each facet, we state if we obtain a *p*<0.05 (Wald’s test) for significant growth inhibition after 1.5 h in M*e*^x^.


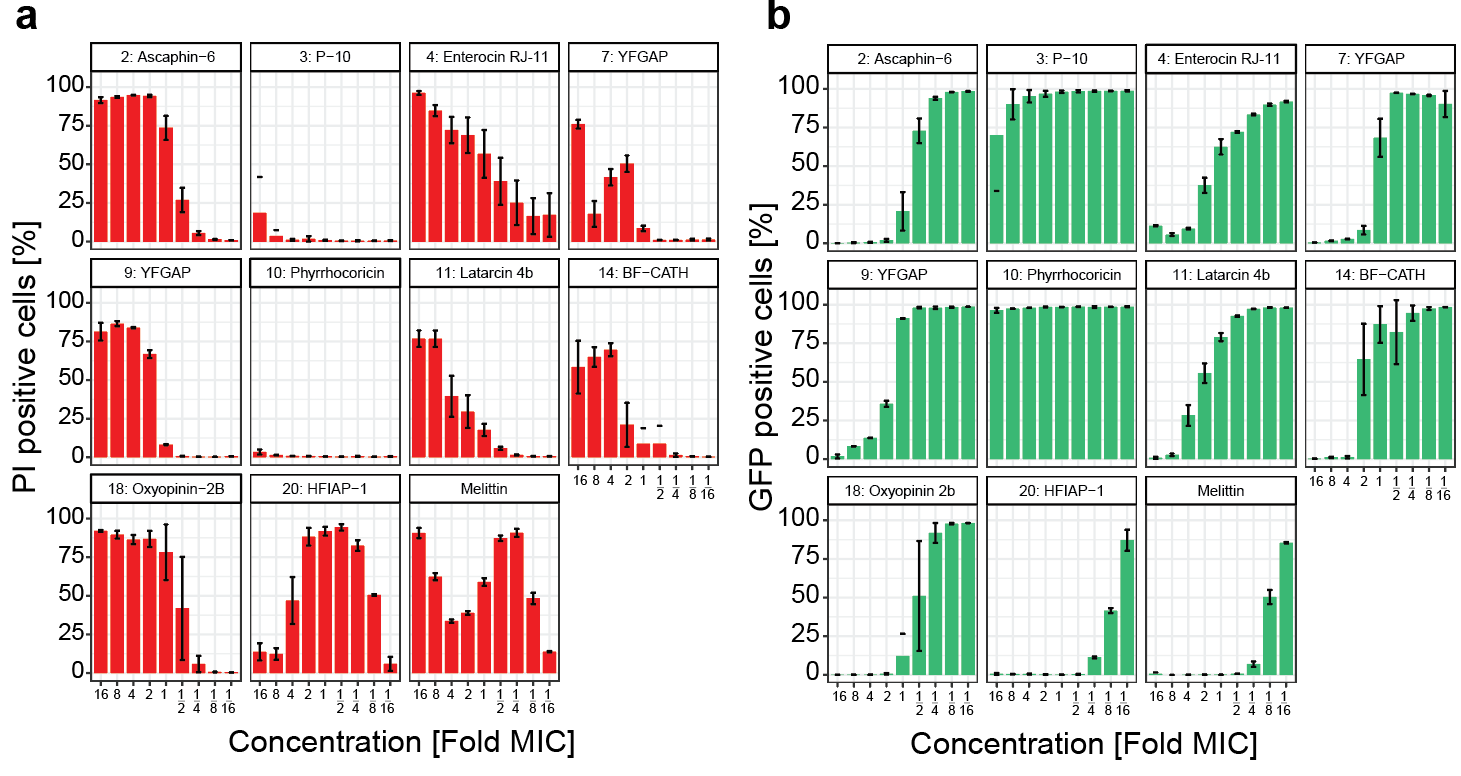


**Figure S14 | Membrane damage assay of chemically purified peptides.** Membrane damage is assessed for all peptides that proved to be active in MIC assays by measuring **(a)** PI uptake of the cells (% cells are given that gained PI fluorescence) and **(b)** GFP loss (% of the cells are given that lost GFP fluorescence), when incubating *E. coli* TOP10 cells (n=2, error bars: 4σ) with increasing concentrations of chemically synthesized peptides starting from MIC/16 to 16xMIC in 2-fold dilutions. GFP loss indicated massive damage of both outer and cytoplasmic (inner) membrane, whereas PI staining is only indicative for damage of the inner but not for outer membrane, which can be traversed by the dye even in intact cells. The highly membrane damaging peptide Melittin is used as a control. Note: at concentration higher than 4xMIC (16 µM), Melittin started to precipitate.

**
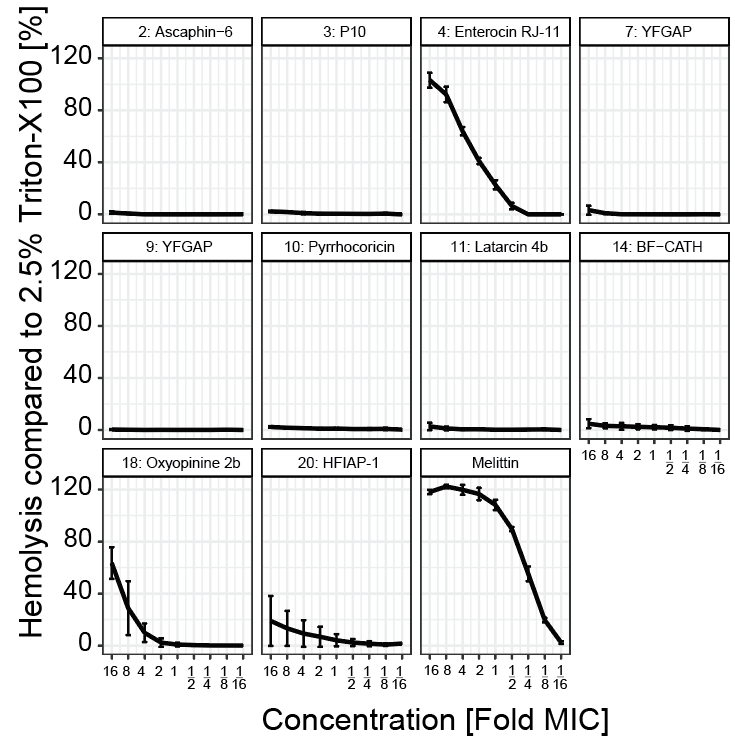
**

**Figure S15 | Hemolytic activity of chemically purified peptides.** Assessment of the hemolytic power of all peptides that proved to be active in MIC assays. Hemolysis is assessed incubating human erythrocytes (n=4, error bars: 4σ) with increasing peptide concentrations starting from MIC/16 to 16xMIC in 2-fold dilutions. Hemolysis in % is compared to detergent-treated (2.5% Triton-X100) erythrocytes

**Table S1 |** Additional information on 20 most active peptides in M*e*^x^.*by OD_ID_ (4.5h)

| Rank* | parent name | Sequence | Origin | ID | Previously reported activity against *E.coli* of parent | Reported antibacterial mechanism of action of parent | Original reference |
| --- | --- | --- | --- | --- | --- | --- | --- |
| 1 | Meucin-25 | VKLIQIRIWIQYVTVLQMFSMKTKQ | APD | 11598 | not active | none | ^1^ |
| 2 | Ascaphin-6 | GFKDWIKGAAKKLIKTVASSIANE | APD | 9286 | active | Membrane damage | ^2^ |
| 3 | P-10 | VSKIKKYLKYKDRI | APD | 8942 | active | none | ^3^ |
| 4 | Enterocin RJ-11 | AIAKLVAKFGWPIVKKYYKQIMQFIGEGWAINKIIEWIKK | NCBI | 3780 | not determined | Membrane damage | ^4^ |
| 5 | PepG1 | MITISTMLQFGLFLIALIGLVIKLIELSIKK | NCBI | 11834 | not active | Membrane damage | ^5^ |
| 6 | PepG1 | LVTLSAMLQFGIFLIAFIGLVIDLIKLSQKK | NCBI | 11828 | not active | Membrane damage | ^5^ |
| 7 | YFGAP | VKVGINGFGRIGRLVTRAAFQSKKVEIVAIND | NCBI | 8112 | active | None-membrane damaging | ^6^ |
| 8 | PepG1 | MITISTMLQFGLFLIALIGLVIKLIELSNK | NCBI | 11833 | not active | Membrane damage | ^5^ |
| 9 | YFGAP | VKVGVNGFGRIGRLVTRAAFNSGKVEIVAIND | NCBI | 8135 | active | None-membrane damaging | ^6^ |
| 10 | Pyrrhocoricin | VDKGGYLPRPTPPRPVYR | NCBI | 7122 | active | Translation inhibition | ^7^ |
| 11 | Latarcin 4a | LKDKVKSMGEKLKQYIQTWKAKF | APD | 5147 | active | None-membrane damaging | ^8^ |
| 12 | PepG1 | LVYISIMLQFGMFIIAFIGLVIELIKLRQK | NCBI | 11836 | not active | Membrane damage | ^5^ |
| 13 | Vv-AMP1 | RACESQSHRFKGTCVRQSNCAAVCQTE | NCBI | 8053 | not active | none | ^9^ |
| 14 | BF-CATH | KRFKKFFKKLKKSVKKRAKKFFKKPRVIGVSIPF | APD | 9639 | active | none | ^10^ |
| 15 | Delta lysin I | MAADIISTIGDLVKWIIDTVNKFK | NCBI | 3458 | not active | Membrane damage | ^11^ |
| 16 | Maximin 3 | TALKGAAKELASTYQH | NCBI | 5468 | active | Membrane damage | ^12^ |
| 17 | PepG1 | PMLQFGLFLIALIGLVIKLIELSNKK | NCBI | 11827 | not active | Membrane damage | ^5^ |
| 18 | Oxyopinin 2b | GKFSGFAKILKSIAKFFKGVGKVRKGFKEASDLDKNQ | APD | 9690 | active | Membrane damage | ^13^ |
| 19 | Cycloviolacin H2 | SYIPCGESCVYIPCTVTALLGCSCSNKVCYKN | NCBI | 10889 | not active | none | ^14^ |
| 20 | HFIAP-1 | GWFKKAWRKVKHAGRRVLDTAKGVGRHYLNNWLNRYR | NCBI | 4545 | active | Membrane damage | ^15^ |

| Strains | ATCC | MIC [µm] in MHB |
| --- | --- | --- |
| *Staphylococcus aureus* | 29213 | 1.4 |
| *Pseudomonas aeruginosa* | 47085 | 0.7 |
| *Enterococcus faecalis* | 29212 | 5.6 |
| *Klebsiella pneumonia* | 13883 | 0.4 |
| *E. coli* | 25922 | 0.7 |

**Table S2 |** Antimicrobial activity of HFIAP-1_NCBI 4545_. Mean MIC-values are recorded (n=3) in microtiter plate assays using chemically synthesized peptide.

**Bibliography of Supplement**

1. Gao, B. *et al.* Characterization of two linear cationic antimalarial peptides in the scorpion Mesobuthus eupeus. *Biochimie* **92,** 350–359 (2010).

2. Conlon, J. M., Sonnevend, A., Davidson, C., David Smith, D. & Nielsen, P. F. The ascaphins: A family of antimicrobial peptides from the skin secretions of the most primitive extant frog, Ascaphus truei. *Biochem. Biophys. Res. Commun.* **320,** 170–175 (2004).

3. Lu, Y., Zhuang, Y. & Liu, J. Mining antimicrobial peptides from small open reading frames in Ciona intestinalis. *J. Pept. Sci.* **20,** 25–29 (2014).

4. Cintas, L. M. *et al.* Enterocins L50A and L50B, two novel bacteriocins from Enterococcus faecium L50, are related to staphylococcal hemolysins. *J. Bacteriol.* **180,** 1988–1994 (1998).

5. Felden, B. Dual Toxic-Peptide-Coding Staphylococcus aureus RNA under Antisense Regulation Targets Host Cells and Bacterial Rivals Unequally. *Cell Rep.* **7,** 424–435 (2014).

6. Seo, J. K., Lee, M. J., Go, H. J., Park, T. H. & Park, N. G. Purification and characterization of YFGAP, a GAPDH-related novel antimicrobial peptide, from the skin of yellowfin tuna, Thunnus albacares. *Fish Shellfish Immunol.* **33,** 743–752 (2012).

7. Chernysh, S., Cociancich, S., Briand, J. P., Hetru, C. & Bulet, P. The inducible antibacterial peptides of the hemipteran insect Palomena prasina: Identification of a unique family of proline-rich peptides and of a novel insect defensin. *J. Insect Physiol.* **42,** 81–89 (1996).

8. Kozlov, S. A. *et al.* Latarcins, antimicrobial and cytolytic peptides from the venom of the spider Lachesana tarabaevi (Zodariidae) that exemplify biomolecular diversity. *J. Biol. Chem.* **281,** 20983–20992 (2006).

9. De Beer, A. & Vivier, M. A. Vv-AMP1, a ripening induced peptide from Vitis vinifera shows strong antifungal activity. *BMC Plant Biol.* **8,** 1–16 (2008).

10. Zhao, H. *et al.* Identification and characterization of novel reptile cathelicidins from elapid snakes. *Peptides* **29,** 1685–1691 (2008).

11. Verdon, J., Berjeaud, J. M., Lacombe, C. & Héchard, Y. Characterization of anti-Legionella activity of warnericin RK and delta-lysin I from Staphylococcus warneri. *Peptides* **29,** 978–984 (2008).

12. Lai, R. *et al.* Antimicrobial peptides from skin secretions of Chinese red belly toad Bombina maxima. *Peptides* **23,** 427–435 (2002).

13. Corzo, G. *et al.* Oxyopinins, large amphipathic peptides isolated from the venom of the wolf spider Oxyopes kitabensis with cytolytic properties and positive insecticidal cooperativity with spider neurotoxins. *J. Biol. Chem.* **277,** 23627–23637 (2002).

14. Chen, B. *et al.* Isolation and characterization of novel cyclotides from Viola hederaceae: Solution structure and anti-HIV activity of vhl-1, a leaf-specific expressed cyclotide. *J. Biol. Chem.* **280,** 22395–22405 (2005).

15. Uzzell, T., Stolzenberg, E. D., Shinnar, A. E. & Zasloff, M. Hagfish intestinal antimicrobial peptides are ancient cathelicidins. *Peptides* **24,** 1655–1667 (2003).
